# Computational pharmacovigilance of tranexamic acid: implications for intracerebral hemorrhage based on FAERS database and network toxicology

**DOI:** 10.3389/fphar.2026.1809788

**Published:** 2026-06-09

**Authors:** Jia-Wei Wu, Cui-Cui Shen, Peng Wang, Bing-Xin Wang, Cui-Yan Chen, Zeng-Li Miao, Xu-Dong Zhao, Wei Gao

**Affiliations:** 1 Department of Neurosurgery, Jiangnan University Medical Center, Wuxi, Jiangsu, China; 2 Wuxi School of Medicine, Jiangnan University, Wuxi, Jiangsu, China; 3 Department of Acupuncture and Moxibustion, Jiangnan University Medical Center, Wuxi, Jiangsu, China; 4 Department of Neurosurgery, Medical School of Nantong University, Nantong University, Nantong, Jiangsu, China; 5 Department of Neurology, Wuxi Ninth People’s Hospital Affiliated to Soochow University, Wuxi, Jiangsu, China

**Keywords:** adverse drug events, intracerebral hemorrhage, molecular docking, network toxicology, tranexamic acid

## Abstract

**Background:**

Tranexamic acid (TXA) is a widely used antifibrinolytic agent for the management of hemorrhagic disorders and has increasingly been investigated for the treatment of intracerebral hemorrhage (ICH). However, TXA-associated adverse drug events (ADEs), particularly neurological complications, remain insufficiently characterized, despite their association with unfavorable neurological outcomes, prolonged hospitalization, and increased in-hospital mortality. A systematic evaluation of TXA-related safety risks and underlying mechanisms in ICH is warranted.

**Materials and methods:**

FAERS reports from the first quarter of 2004 to the third quarter of 2023 were analyzed to identify TXA-associated ADE signals using a hierarchical prediction framework integrating statistical learning algorithms. Network toxicology analyses were conducted to explore potential molecular mechanisms and key regulatory targets involved in TXA-related effects in ICH. Drug–target interactions were further assessed by molecular docking and molecular dynamics simulations. *In vitro* experiments using ICH-related cell models were performed to validate the predicted neurotoxic effects of TXA.

**Results:**

Eight significant neurologically related ADE signals associated with TXA were identified, including myoclonic seizures and status epilepticus. Network toxicology analysis highlighted CASP3, PPARA, and BCL2 as key regulatory genes potentially mediating TXA-related neurotoxicity in ICH. Molecular docking demonstrated strong binding affinities between TXA and core targets, with binding energies lower than −8.0 kcal/mol. Molecular dynamics simulations confirmed stable binding conformations, with root mean square deviation values below 2.0 Å. *In vitro* experiments further supported the potential neurotoxic effects of TXA in ICH cell models.

**Conclusion:**

This study establishes an integrated computational-to-experimental framework for evaluating the safety of TXA in ICH. The FAERS analysis revealed overall neurological ADE signals for TXA. Combined with network toxicology and *in vitro* data, these findings suggest potential neurotoxic mechanisms that warrant cautious evaluation in future ICH-specific studies.

## 1 Introduction

Intracerebral hemorrhage (ICH) is a major cause of disability and mortality among stroke patients, imposing substantial economic and emotional burdens on families and society (Wu et al., 2025a). In addition to non-modifiable prognostic factors such as age and preexisting conditions, hematoma volume is a critical determinant of clinical outcome (Wei et al., 2025; Liu Y. et al., 2025). Of particular importance is the dynamic evolution of hemorrhage volume during the early phase after onset, especially within the first few hours (Du et al., 2026). Clinical studies indicate that approximately one-third of patients presenting within 3–6 h experience hematoma expansion, a process strongly associated with neurological deterioration and poor prognosis (Veltkamp and Purrucker, 2017). Consequently, preventing hematoma expansion has emerged as a central goal of early ICH management (Wu et al., 2024). Over recent decades, numerous medical and surgical strategies for ICH treatment have been investigated. Accumulating evidence demonstrates that, similar to ischemic stroke, specialized care provided by multidisciplinary stroke teams or neurocritical care units significantly improves patient outcomes (Davis et al., 2025). Nevertheless, despite validation through large-scale clinical trials, current guidelines continue to highlight substantial uncertainties in ICH diagnosis and management. Moreover, increasing evidence suggests that some commonly used interventions may be ineffective or even harmful and should be avoided.

As an indirect systemic fibrinolytic inhibitor, tranexamic acid (TXA) has been widely used since its introduction in the 1960s (Rohwer et al., 2025), with its clinical indications steadily expanding in recent years. The landmark CRASH-2 trial in 2010 brought TXA to international prominence, demonstrating a 16.7% reduction in bleeding-related mortality and an approximately 10% decrease in overall mortality (Shakur et al., 2010). Subsequently, the World Health Organization listed TXA as an essential treatment for acute bleeding conditions, including trauma, cardiopulmonary bypass, and postpartum hemorrhage (Tian et al., 2024). In 2013, European guidelines further recommended prophylactic TXA use during major surgery to reduce perioperative blood loss and transfusion requirements (Kozek-Langenecker et al., 2013). Given its proven efficacy in hemorrhage control and the critical prognostic role of hematoma volume in ICH, multiple clinical trials have evaluated TXA in acute ICH. However, most available evidence suggests that TXA does not confer meaningful clinical benefit in this setting (Hollingworth et al., 2024; Polymeris et al., 2023), despite the continued proliferation of related studies. More importantly, as TXA use increases and its application in ICH expands, concerns regarding potential adverse effects, including thromboembolic complications, seizures, and increased mortality, have become increasingly prominent (Nishida et al., 2017; Chen et al., 2026). Therefore, a comprehensive assessment of TXA safety in ICH treatment is urgently needed before pursuing further clinical trials.

This study systematically evaluated the safety profile of TXA using real-world pharmacovigilance data from the U.S. Food and Drug Administration Adverse Event Reporting System (FAERS). Potential targets and molecular mechanisms underlying TXA-associated toxicity in ICH were further explored through an integrated approach combining network toxicology, molecular docking, and *in vitro* validation. Network toxicology, as an emerging systems-level method, enables comprehensive analysis of compound-related toxicological mechanisms, while molecular docking predicts ligand–receptor interactions by estimating binding energies; lower energies indicate greater binding stability. By integrating FAERS signal mining, network toxicology prediction, and experimental validation, this study aims to characterize TXA-related adverse neurological events, identify key toxicity-associated targets in ICH, and elucidate relevant molecular pathways. It should be noted that the FAERS-based signal detection captures ADEs from TXA used across all approved indications (e.g., trauma, postpartum hemorrhage, surgery), not exclusively in ICH patients. The subsequent network toxicology and *in vitro* experiments were therefore designed as a hypothesis-generating investigation of whether the observed neurological signals could be mechanistically linked to ICH pathophysiology. Overall, these findings are expected to clarify the safety characteristics of TXA in ICH treatment, identify potential risk factors, and provide mechanistic evidence to support clinical decision-making.

## 2 Materials and methods

### 2.1 Reagents

The chemical reagents used in this study include: tranexamic acid (TXA, HY-B0149), dimethyl sulfoxide (DMSO, HY-Y0320D), and hemin (HY-19424), all procured from MedCheminxpress (Shanghai). Dulbecco’s modified Eagle’s medium (DMEM, high glucose; PM150210), fetal bovine serum (FBS; 164,210), 100× penicillin–streptomycin solution (PB180120), and 0.25% trypsin–EDTA (PB180227, in D-Hank’s solution) were supplied by ProNova Life Science Technology Co., Ltd. (Wuhan). The Enhanced CCK-8 Detection Kit (C0043) was provided by Biyuntian Biotechnology Co., Ltd. (Shanghai).

### 2.2 Cells

HT22 hippocampal neuronal cells (CL-0697) and BV2 microglial cells (CL-0493) were purchased from Wuhan Procell Life Science & Technology Co., Ltd. Cells were maintained in DMEM supplemented with 10% heat-inactivated FBS and 1% penicillin–streptomycin at 37 °C in a humidified incubator containing 5% CO_2_. TXA and hemin were dissolved in DMSO to prepare stock solutions, which were diluted with complete medium immediately before use; the final DMSO concentration did not exceed 0.1%. To establish an *in vitro* ICH model, cells were serum-starved for 12 h and then exposed to 50 μM hemin for 12 h, as previously described (Wu et al., 2025b).

### 2.3 Data extraction

Time-series pharmacovigilance data were extracted from the U.S. FAERS database spanning the first quarter (Q1) of 2004 to the third quarter (Q3) of 2023, covering the entire post-marketing period of tranexamic acid (TXA). Data cleaning and deduplication were performed using R software (version 4.3.1). For each unique case identifier (CASEID), the report with the most recent FDA_DT was retained; if two reports had the same FDA_DT, the report with the higher ISR number was kept. Drug names were standardized using the MedEx_UIMA system (version 1.8.3) (Liang et al., 2024) and further mapped to RxNorm terminology to unify all synonyms and brand names of tranexamic acid. Only reports listing “tranexamic acid” as the drug name and classified as “Primary Suspect (PS)” were included in the primary analysis. Datasets from different files (e.g., DEMO, DRUG, REAC, OUTC) were linked using the primaryid field. For missing data in categorical variables such as sex, age group, and reporter type, no imputation was performed; instead, a separate “Missing” category was created and reported in the demographic summary to transparently present the extent of missing information.

### 2.4 Data analysis

A comprehensive signal detection strategy was implemented using four complementary disproportionality analysis methods: reporting odds ratio (ROR), proportional reporting ratio (PRR), Bayesian confidence propagation neural network (BCPNN) (Shu et al., 2025), and empirical Bayesian geometric mean (EBGM) (Li et al., 2026) (Table 1). For each adverse event (AE) of interest, a standard 2 × 2 contingency table was constructed as follows.

**Table:** 

Title	Target AE	All other AEs	Total
TXA	a	b	a + b
All other drugs	c	d	c + d
Total	a + c	b + d	N

**TABLE 1:** Disproportionality analysis of tranexamic acid-associated adverse event signals at the preferred term level.

Preferred term (PT)	a	ROR (95% CI)	PRR (χ^2^)	IC (IC-2SD)	EBGM (EB05)
Myoclonic seizure	17	174.12 (107.72–281.45)	173.82 (2734.5)	6.23 (5.89)	78.4 (52.1)
Myoclonus	71	82.38 (64.99–104.43)	81.56 (5298.2)	5.43 (5.12)	42.9 (34.8)
Status epilepticus	43	53.68 (39.72–72.54)	53.21 (2076.1)	4.97 (4.61)	30.5 (23.4)
Cerebral artery thrombosis	5	100.36 (41.56–242.36)	100.28 (456.3)	5.18 (4.02)	35.6 (18.9)
Dystonic tremor	8	45.23 (22.56–90.69)	45.18 (326.8)	4.78 (4.01)	27.4 (15.2)
Generalized tonic-clonic seizure	12	32.15 (18.21–56.74)	32.08 (354.2)	4.51 (3.92)	22.8 (13.9)
Cerebral infarction	9	6.78 (3.52–13.06)	6.76 (48.2)	2.68 (1.95)	6.4 (3.8)
Deep vein thrombosis	15	4.23 (2.55–7.01)	4.22 (38.9)	2.07 (1.48)	4.2 (2.7)
Pulmonary embolism	11	3.98 (2.20–7.20)	3.97 (26.5)	1.99 (1.32)	4.0 (2.4)
Acute kidney injury	21	2.45 (1.60–3.76)	2.45 (15.8)	1.29 (0.71)	2.5 (1.7)

Number of TXA, reports (a); The full list of all PTs, meeting signal criteria including those with lower reporting frequency is provided in Supplementary Table S3. All thresholds: ROR, 95% CI, lower limit >1; PRR ≥2 and χ^2^ ≥ 4; IC-2SD > 0; EB05 > 2*.

The definitions and signal thresholds for each algorithm were:

ROR = (a/c)/(b/d). A signal was considered present when the number of reports with TXA and the target AE (a) ≥ 3 and the lower limit of the 95% confidence interval of ROR > 1.

PRR = [a/(a + b)]/[c/(c + d)]. A signal was defined as a ≥ 3, PRR ≥ 2, and the chi-square statistic (χ^2^) ≥ 4.

BCPNN: The information component (IC) was calculated as IC = log_2_ [a(a + b + c + d)]/[(a + b) (a + c)]. A positive signal was indicated when the lower limit of the 95% confidence interval of IC (IC – 2SD) > 0.

EBGM: The empirical Bayesian geometric mean was computed using the Gamma-Poisson shrinker model. A signal was declared when EB05 > 2 (i.e., the lower bound of the 95% confidence interval for EBGM exceeds 2).

To reduce false-positive signals, a multi-algorithm validation approach was adopted: only those AE terms that met the signal criteria in at least three of the four methods were considered statistically robust signals for further analysis. Detailed calculation formulas and signal detection thresholds are provided in the Supplementary Material. All statistical analyses were performed using R software (version 4.3.1) with the phm and openFDA packages.

#### 2.4.1 Sensitivity and stratified analyses

To evaluate the robustness of the primary signal detection results and to explore potential effect modification, a series of sensitivity and stratified analyses were performed.

Sensitivity analyses: Restriction to serious reports: All disproportionality analyses were repeated using only adverse event reports classified as “serious” (including outcomes such as death, life-threatening, hospitalization, disability, congenital anomaly, or intervention required to prevent permanent impairment). Restriction to healthcare professional reports: Analyses were limited to reports submitted by physicians, pharmacists, and other healthcare professionals to assess the potential impact of reporter type on signal estimates. Restriction to primary suspect (PS) role only: This was the primary analysis (already described); additionally, an exploratory analysis including reports with TXA as “secondary suspect (SS)” or “concomitant (C)” was performed to assess the influence of suspect role assignment.

Stratified analyses: By sex: Signal detection was performed separately for female and male subgroups to identify potential sex-specific adverse event patterns. By age group: Reports were stratified into adults (18–64 years) and elderly (≥65 years). The age subgroup with the highest reporting frequency was used as the reference for comparison. By time period: The entire study period was divided into two eras: early (2004–2013) and late (2014–2023), to evaluate temporal stability of the detected signals. For each sensitivity and stratified analysis, the same signal detection thresholds (a ≥ 3, and at least three out of four algorithms positive) were applied. Results were compared with the primary analysis to assess consistency. All analyses were conducted using R software (version 4.3.1).

### 2.5 Signal filtering and categorization

Adverse event signals were initially filtered to retain only those with a reporting frequency (a) ≥ 3. All retained Preferred Terms (PTs) were then standardized and hierarchically classified according to the Medical Dictionary for Regulatory Activities (MedDRA, version 26.0). Each PT was mapped to its corresponding System Organ Class (SOC) and, where applicable, to Standardised MedDRA Queries (SMQs) for neurological and thromboembolic events. The distribution of signals across SOCs was analyzed to identify organ systems disproportionately associated with TXA (Lin et al., 2025). Detailed contingency tables and all four disproportionality metrics (ROR, PRR, IC, EBGM) for each PT are provided in the Supplementary Materials (Supplementary Tables S2, S3).

### 2.6 Collection of TXA and ICH-related targets

Potential human targets of TXA were retrieved from the SwissTargetPrediction (http://www.swisstargetprediction.ch/), ChEMBL (https://www.ebi.ac.uk/chembl/), and STITCH databases (http://stitch.embl.de/) (Table 2). Using “intracranial hemorrhage” and “cerebral hemorrhage” as search terms, ICH-related genes were obtained from the GeneCards gene database (https://www.genecards.org/) and the OMIM genetic disease database (https://omim.org/) (Wu et al., 2025c). The gene sets were deduplicated and standardized using the UniProt protein database (https://www.uniprot.org/) to identify common targets between TXA and ICH.

**TABLE 2:** TXA basic information.

Formula	Molecular weight	Gi absorption	BBB permeant	tPSA	Log *K*_p_ (skin permeation)
C_7_H_13_NO_2_	143.18 g/mol	High	Yes	63.32 Å^2^	−8.73 cm/s

### 2.7 Construction of protein–protein interaction (PPI) network

Using the STRING interaction database (https://cn.string-db.org/) and “*Homo sapiens*” species parameters (Du et al., 2025), a PPI network was constructed based on the intersection of TXA targets and ICH-related genes. A high confidence threshold (interaction score > 0.7) was set. The STRING output data were imported into Cytoscape analysis software (v3.10.1) for network topology feature evaluation (Table 3). Core targets were identified using the maximal clique centrality (MCC) algorithm implemented in the CytoHubba plugin. To further elucidate functional relationships among the top ten key targets, interaction networks were analyzed using the GeneMANIA platform (https://genemania.org/). Modular analysis performed with the MCODE plugin revealed that four core targets were centrally located within the most significant functional modules.

**TABLE 3:** Information about the top 10 PPI networks.

Name	Average shortest path length	Betweenness centrality	Closeness centrality	Degree
CASP3	1.679	0.262	0.595	44
PPARA	1.886	0.141	0.530	36
BCL2	1.830	0.097	0.546	34
REN	1.905	0.077	0.524	30
CYP3A4	1.924	0.087	0.519	28
CYP19A1	2.000	0.091	0.500	24
NR3C1	2.094	0.025	0.477	22
ANPEP	2.320	0.053	0.430	20
TIMP1	2.075	0.037	0.481	20
PTPRC	2.169	0.028	0.460	20

### 2.8 Gene function and pathway enrichment analysis of the target protein

The Metascape network platform (https://metascape.org/) was used to conduct a comprehensive analysis of shared target genes between TXA and ICH. Gene Ontology (GO) analysis was conducted to systematically evaluate biological processes (BP), cellular components (CC), and molecular functions (MF), thereby elucidating the biological roles of the shared targets (Yu et al., 2023). Kyoto Encyclopedia of Genes and Genomes (KEGG) pathway enrichment analysis was further performed to identify treatment-related signaling pathways and potential toxicity-associated pathways, facilitating target prioritization and mechanistic interpretation of TXA action. Enrichment results were visualized using the MicroBioinformatics online platform (https://www.bioinformatics.com.cn/).

### 2.9 Molecular docking and molecular dynamics simulation of TXA with core targets

Three-dimensional structural data for key target proteins were sourced from the Protein Data Bank (RCSB PDB, http://www.pdb.org/) and preprocessed using AutoDock software (v4.2.6) (Zhang et al., 2025). Molecular docking was performed using AutoDock Vina, and docking conformations were visualized with PyMOL (version 2.4.1). A root-mean-square deviation (RMSD) ≤ 2.0 Å was used as the criterion for validating docking reliability. Molecular dynamics (MD) simulations of TXA–protein complexes were conducted using Discovery Studio 2019 (DS) (Riyaphan et al., 2021). Simulations were run for 100 ns with the Complex. dsv solvent system enabled. Trajectories were monitored using the Job Browser module, and DS built-in analysis tools were used to evaluate dynamic hydrogen bond interactions, as well as RMSD and RMSF values of protein backbone and side chains.

### 2.10 CCK-8 assay

Cell viability was assessed using the CCK-8 assay as previously described (Liu S. et al., 2025). Cells were seeded into 96-well plates at a density of 5 × 10^3^ cells per well and treated with gradient concentrations of TXA or vehicle control (DMSO) for 12, 24, and 48 h. Subsequently, 10 μL of CCK-8 reagent was added to each well, followed by incubation at 37 °C in a 5% CO_2_ incubator for 2 h. Absorbance at 450 nm was measured using a Thermo Fisher Scientific microplate reader. All experiments were performed with three independent biological replicates.

### 2.11 Western blot (WB)

Total protein was extracted from cells using RIPA lysis buffer, and protein concentrations were determined by the bicinchoninic acid (BCA) assay (Xue et al., 2025). Equal amounts of protein were separated by sodium dodecyl sulfate–polyacrylamide gel electrophoresis (SDS–PAGE) and transferred onto polyvinylidene difluoride (PVDF) membranes (Millipore, MA, USA). Membranes were blocked with 5% bovine serum albumin (BSA) at room temperature for 1–2 h and then incubated with primary antibodies at 4 °C overnight. After incubation with horseradish peroxidase (HRP)-conjugated secondary antibodies at room temperature for 1–2 h, immunoreactive bands were visualized using an enhanced chemiluminescence (ECL) detection system (Millipore). Band intensities were quantified using ImageJ software (version 1.5, NIH).

### 2.12 Immunofluorescence assay

Cells were seeded onto coverslips in 24-well plates at a density of 3 × 10^4^ cells per well and cultured for 12 h (Li et al., 2024). Following TXA treatment, cells were washed three times with ice-cold phosphate-buffered saline (PBS) and fixed with 4% paraformaldehyde for 10–20 min. Cells were then permeabilized with 0.5% Triton X-100 for 10–15 min and blocked with 10% BSA for 1–2 h. Primary antibodies (1:150 dilution) were applied and incubated overnight at 4 °C. After incubation with fluorescently labeled secondary antibodies at room temperature for 1–2 h, nuclei were counterstained with DAPI (C0065, Solarbio, Beijing) for 15–20 min. Coverslips were mounted, and images were captured using a Zeiss fluorescence microscope (Axio Imager 2, Germany).

### 2.13 RNA isolation and qPCR

Total RNA extraction was performed using Trizol reagent (Qiagen, Germany) (Guo et al., 2025). Reverse transcription was performed with the 5× All-in-One RT PreMix Kit (R333-01, Vazyme, China). Quantitative real-time PCR (qPCR) was carried out using SYBR Green qPCR Master Mix (Q711-02, Vazyme, China), with six technical replicates per group. GAPDH served as the internal reference gene for expression normalization. Relative expression levels were calculated using the 2^−ΔΔCt^ method. Primer information is listed in Table 4.

**TABLE 4:** List of primers used for RT-qPCR.

Gene	Forward (5′–3′)	Reverse (5′–3′)	Number of bases
Mouse-MMP2	CAA​GGA​TGG​ACT​CCT​GGC​ACA​T	TAC​TCG​CCA​TCA​GCG​TTC​CCA​T	22/22
Mouse-MMP3	CTC​TGG​AAC​CTG​AGA​CAT​CAC​C	AGG​AGT​CCT​GAG​AGA​TTT​GCG	22/21
Mouse-MMP9	GCT​GAC​TAC​GAT​AAG​GAC​GGC​A	TAG​TGG​TGC​AGG​CAG​AGT​AGG​A	22/22
Mouse-GAPDH	AGG​TCG​GTG​TGA​ACG​GAT​TTG	GGG​GTC​GTT​GAT​GGC​AAC​A	21/19

### 2.14 Cellular thermal shift assay (CETSA) and drug affinity‐responsive target stability (DARTS) assay

The CETSA assay was performed according to a previously established protocol (Zhang et al., 2026). 100 μL of HT22 cell lysate was treated with 25 μM TXA, followed by incubation in a temperature gradient ranging from 35 °C to 71 °C, with a 3-min increase in temperature at each step. After heating, the samples were centrifuged at 20,000 g for 10 min at 4 °C. The supernatant was collected, 5× loading buffer was added, and Western blotting analysis was performed following 10% SDS-PAGE electrophoresis. DARTS experiments were performed according to previously described methods (Xu et al., 2026). HT22 cell lysates were incubated with various concentrations of TXA at room temperature for 1 h. Pronase E (5 μg/mL; HY-114158, MedChemExpress, USA) was then added, and digestion was carried out for 45 min. The reaction was terminated by adding loading buffer, and the protein samples were subsequently analyzed by Western blot.

## 3 Statistical analysis

All statistical analyses were performed using GraphPad Prism software (version 10.0.2; GraphPad Software, La Jolla, CA, USA). Data are presented as mean ± standard deviation (SD) from at least three independent experiments (n ≥ 3). Data normality was assessed prior to statistical testing. For normally distributed data, two-way analysis of variance (ANOVA) followed by Tukey’s *post hoc* test was applied to assess differences between groups. Statistical significance was defined as *P < 0.05, **P < 0.01, ***P < 0.001, and #P < 0.0001. No samples were excluded, and no outliers were removed during data analysis.

## 4 Results

### 4.1 Dataset characteristic analysis

#### 4.1.1 Study population demographics and reporting patterns

To characterize TXA-related adverse drug events (ADEs), demographic and temporal analyses were performed on reports extracted from the FAERS database between Q1 2004 and Q3 2023. A total of 1,577 TXA-associated ADE reports were identified. Detailed demographic characteristics are summarized in Supplementary Tables S1. Among reports with available sex information, females accounted for 53.01% (n = 836), whereas males represented 29.74% (n = 469), yielding a female-to-male ratio of 1.78:1 (Supplementary Figure S1A). A notable proportion of reports lacked sex information (17.24%, n = 272). Age distribution showed that most reports involved adults aged 18–64 years (41.79%, n = 659) and individuals aged ≥65 years (26.57%, n = 419). However, age data were missing in 25.23% of cases (n = 398), limiting age-stratified analyses (Supplementary Figure S1B). Regarding routes of administration, intravenous use was reported in 26.57% of cases (n = 419), while oral administration accounted for 16.29% (n = 257). More than half of the reports (51.87%, n = 818) lacked information on the administration route, limiting further route-specific risk evaluation (Supplementary Figure S1C). Analysis of reporter types indicated that healthcare professionals submitted the majority of reports, with physicians accounting for 24.6% (n = 572), whereas patient-reported cases comprised only 9.83% (n = 155) (Supplementary Figure S1D). Geographically, most reports originated from the United States (32.02%, n = 505), followed by markedly lower contributions from other countries, including India (1.77%) and Spain (1.64%). This distribution reflects the inherent reporting bias of the FAERS database and limits the generalizability of findings to non-U.S. populations.

#### 4.1.2 Temporal trends and weber effect analysis

To examine temporal variations in TXA-related adverse event reporting and assess the presence of a Weber effect, a time-series analysis of FAERS reports from 2004 to 2023 was performed. Temporal trend analysis is a core component of pharmacovigilance, as changes in reporting frequency may reflect true safety signals or reporting biases driven by market dynamics, regulatory actions, or evolving clinical awareness. As shown in Supplementary Figure S2, the annual number of reported ADEs exhibited a typical Weber effect pattern, characterized by an early increase in reporting followed by a gradual decline. Reporting activity remained relatively stable between 2004 and 2009, increased steadily from 2010 to 2018, and reached a peak in 2019. Thereafter, a gradual decrease in reporting was observed through 2023. Overall, this temporal distribution is consistent with post-marketing surveillance patterns, in which reporting intensity increases during periods of heightened clinical attention and subsequently declines as drug use becomes more routine.

### 4.2 Traditional pharmacovigilance signal detection

#### 4.2.1 System organ class level signal analysis

Using established pharmacovigilance methodologies, signal detection was systematically performed across all system organ classes (SOCs). Four complementary disproportionality analysis methods were applied to evaluate adverse event associations, and the consolidated SOC-level results are summarized in Supplementary Table S2. Consistent signals were observed across multiple analytical approaches. At the SOC level, four major categories demonstrated significant associations with TXA-related AEs. Nervous system disorders showed a strong signal, with 787 reported cases and a reporting odds ratio (ROR, 95% CI) of 2.41 (2.23–2.60), indicating a markedly elevated reporting frequency. Vascular disorders also showed a pronounced signal, with 386 cases and an ROR (95% CI) of 4.49 (4.04–4.98), the highest among all SOCs. General disorders and administration site conditions accounted for 437 reports, with an ROR (95% CI) of 0.52 (0.47–0.58), reflecting commonly reported treatment-related reactions such as injection-site discomfort and systemic symptoms. In addition, injuries, poisonings, and procedural complications were identified in 529 cases, with an ROR (95% CI) of 1.42 (1.29–1.55), indicating a moderate but significant association. Overall, SOC-level analysis revealed distinct patterns of TXA-associated AEs, with particularly strong signals observed in neurological and vascular disorder categories.

#### 4.2.2 Preferred term level signal detection and clinical relevance

Preferred term (PT)–level signal detection was performed to identify specific AEs underlying the observed SOC-level associations. This analysis yielded clinically relevant information for safety assessment and monitoring strategies. A total of 10 PTs demonstrated statistically significant signals, all of which were consistently identified across the four disproportionality analysis methods, indicating robust associations between TXA exposure and these AEs. The consolidated results are presented in Supplementary Table S3. Among the detected PTs, neurological AEs predominated. Myoclonus was the most frequently reported event, with 71 cases and a reporting odds ratio (ROR, 95% CI) of 82.38 (64.99–104.43). Status epilepticus also showed a strong signal, with 43 reports and an ROR (95% CI) of 53.68 (39.72–72.54). In addition, myoclonic seizures exhibited the highest signal strength, with 17 cases and an ROR (95% CI) of 174.12 (107.72–281.45). Beyond neurological manifestations, cerebral artery thrombosis emerged as a notable signal, with five reported cases and an ROR of 100.36, indicating a marked disproportionality relative to background reporting rates. Overall, PT-level analysis revealed a clear hierarchy of TXA-associated AEs, dominated by seizure-related neurological outcomes, and highlighted specific safety signals warranting further investigation.

### 4.3 Results of sensitivity and stratified analyses

#### 4.3.1 Sensitivity analyses

When restricting to serious reports (n = 612, 38.8% of total TXA-associated reports), the majority of neurological signals remained statistically significant. The strongest signals for myoclonic seizure (ROR = 152.3, 95% CI: 88.6–261.9) and status epilepticus (ROR = 49.2, 95% CI: 34.1–71.0) were consistent with the primary analysis, albeit with slightly attenuated point estimates. Restriction to healthcare professional reports (n = 1,045, 66.3% of total) yielded similar signal patterns, with no major qualitative differences. When expanding the suspect role to include secondary suspect or concomitant reports, the signals for thromboembolic events (e.g., cerebral artery thrombosis, deep vein thrombosis) became slightly stronger, while neurological signals remained largely unchanged. Detailed results of sensitivity analyses are provided in Supplementary Table S4.

#### 4.3.2 Stratified analyses

By sex: Stratified signal detection by sex showed that neurological signals (myoclonus, status epilepticus) were present in both females and males, but the reporting odds ratios were numerically higher in females (e.g., myoclonus: ROR = 91.2 [68.3–121.8] in females vs. 68.5 [45.2–103.7] in males). Thrombotic signals showed no consistent sex difference. By age group: In the elderly group (≥65 years, n = 419), the signals for status epilepticus and myoclonus were comparable to those in the adult group (18–64 years, n = 659). However, the signal for cerebral artery thrombosis was only detected in the elderly group (ROR = 112.4, 95% CI: 38.9–324.6) and not in the younger adult group. By time period: Comparison between early (2004–2013) and late (2014–2023) periods revealed that most neurological signals were present in both eras, but the reporting frequency and signal intensity for myoclonus and status epilepticus increased substantially after 2014. This temporal pattern may reflect increased clinical awareness and expanded use of TXA in recent years. Summary statistics for all stratified analyses are presented in Supplementary Table S5.

### 4.4 Network toxicology and computational integration analysis

#### 4.4.1 Computational toxicology prediction and molecular insights

**FIGURE 1:**
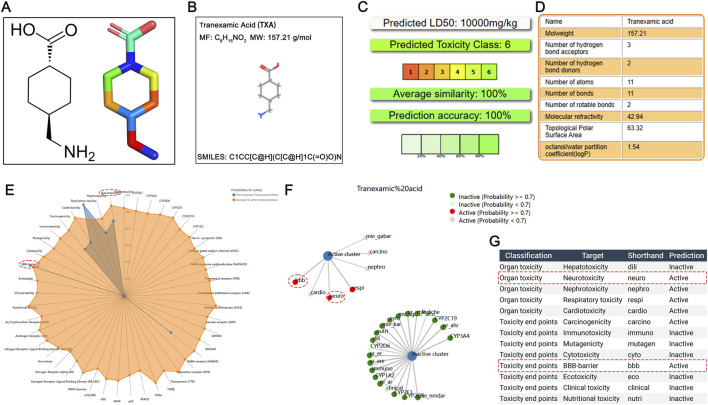
Toxicological prediction results for TXA **(A,B)** SMILES notation and chemical structure of TXA **(C,D)** Toxicity prediction results for TXA indicate an LD50 of 10,000 mg/kg, toxicity level 6, with a predicted confidence level of 100% **(E,F)** TXA exhibits higher toxicity potential for the central nervous system and blood-brain barrier compared to other risk areas.

To explore the potential toxicological properties of TXA, an integrated computational toxicology assessment was performed using multiple online prediction platforms. The overall toxicological evaluation is summarized in Figure 1 (Supplementary Figure S3). The chemical structure of TXA is shown in Figure 1A. Computational prediction estimated an LD_50_ value of 10,000 mg/kg, indicating low acute toxicity. TXA was classified as toxicity level VI, suggesting mild predicted toxicity. The model yielded a predicted confidence level of 100%, reflecting high internal consistency of the computational algorithms and supporting the reliability of the prediction results. Toxicity radar analysis indicated potential associations with neurotoxicity and blood–brain barrier permeability. These predictions provide a mechanistic context for the neurological adverse event signals observed in pharmacovigilance analyses. Overall, the high predicted LD_50_ suggests limited systemic toxicity at therapeutic doses, consistent with the predominance of specific organ-related AEs.

#### 4.4.2 Identification of potential targets

**FIGURE 2:**
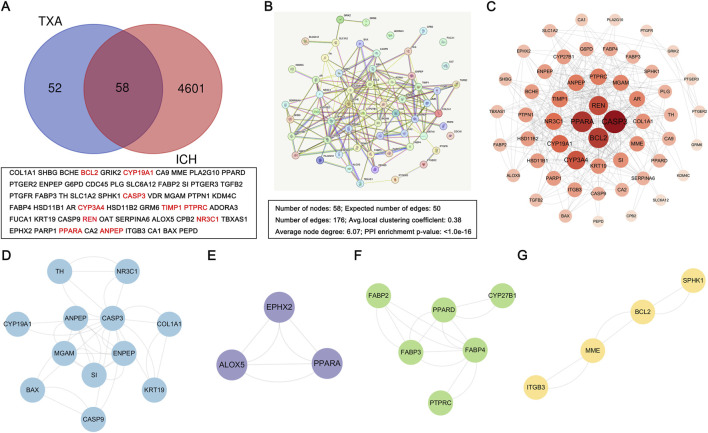
Screening of Key Targets for TXA in ICH Pathogenesis **(A)** Venn diagram and intersecting targets between TXA and ICH targets **(B,C)** PPI network and associated information for intersecting targets **(D–G)** CytoHubba_MCC identifies highly connected PPI subnetworks, with color intensity reflecting weighted scores.

Database screening identified 110 potential human targets associated with TXA and 4,659 genes related to ICH. Intersection analysis identified 58 shared targets, potentially mediating TXA-related effects in ICH (Figure 2A). A PPI network consisting of 58 nodes and 176 edges was constructed using the STRING database (Figure 2B). The network was visualized and topologically analyzed using Cytoscape, with node size and color intensity reflecting degree values (Figure 2C). Using the MCC algorithm implemented in the CytoHubba plugin, seven core targets were identified as key nodes potentially involved in TXA-associated ICH outcomes (Table 5). Further modular analysis using the MCODE plugin revealed multiple functional clusters within the network (Figures 2D–G). Among the core targets, CASP3 had the highest degree, indicating a central position in the interaction network and suggesting a potentially prominent role in TXA-related biological effects.

**TABLE 5:** The binding energy of TXA and Core Targets (kcal/mol).

Target	PDB ID	Structure	Affinity (kcal/mol)	RMSD(Å)
CASP3	1RHM	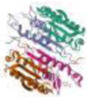	−8.5	1.05
PPARA	3FEI	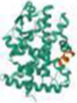	−9.6	1.12
BCL2	5WHH	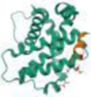	−9.1	1.31
REN	5GT1	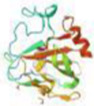	−8.8	1.17
CYP3A4	6UNE	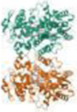	−9.6	1.41
CYP19A1	3S79	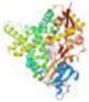	−9.3	1.27
ANPEP	4NAQ	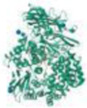	−9.4	1.35

#### 4.4.3 Functional enrichment analysis of potential targets

**FIGURE 3:**
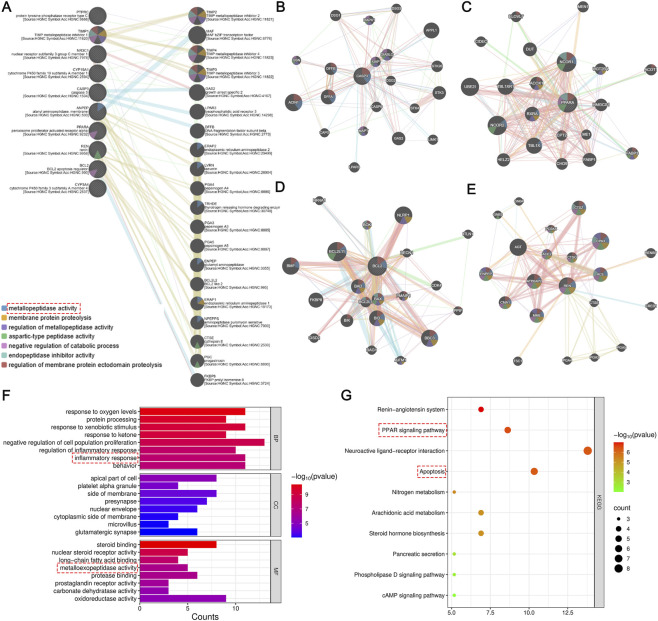
Screening of key mechanism pathways by which TXA influences ICH outcomes **(A)** Functional associations of the top 10 targets by GenMANIA analysis score; functional annotations of target proteins are shown in the lower left corner **(B–E)** Representative GenMANIA images for CASP3, PPARA, BCL2, and REN **(F)** GO enrichment analysis of intersecting targets, including BP, CC, and MF. Highlighting key biological processes, cellular components, and molecular functions potentially affected by TXA exposure **(G)** KEGG enrichment analysis diagram for intersecting targets. Each bar length corresponds to gene count, representing enrichment score and significance level. Higher bars indicate greater counts and higher enrichment.

GeneMANIA analysis identified 20 highly related genes and constructed an interaction network comprising 835 edges. The top ten highly connected genes potentially influenced by TXA in ICH were mainly involved in BP related to metallopeptidase activity, membrane protein proteolysis, and regulation of metallopeptidase activity (Figure 3). To further characterize the functional roles of TXA-associated targets, GO enrichment analysis was performed on the 58 shared targets using the Metascape platform, covering BP, CC, and MF. Enrichment results were ranked by statistical significance and visualized accordingly (Figure 3F). KEGG pathway enrichment analysis identified 41 pathways that were significantly enriched. The top 10 pathways with the highest significance were selected for visualization (Figure 3G). Overall, enrichment analysis indicated that TXA-related targets in ICH are primarily involved in inflammatory responses, are localized to cellular components, including the apical part of the cell, and are associated with molecular functions, including metalloexopeptidase activity. These targets were enriched in multiple signaling pathways, notably the PPAR signaling pathway and apoptosis-related pathways.

#### 4.4.4 Molecular docking of TXA with core target proteins

**FIGURE 4:**
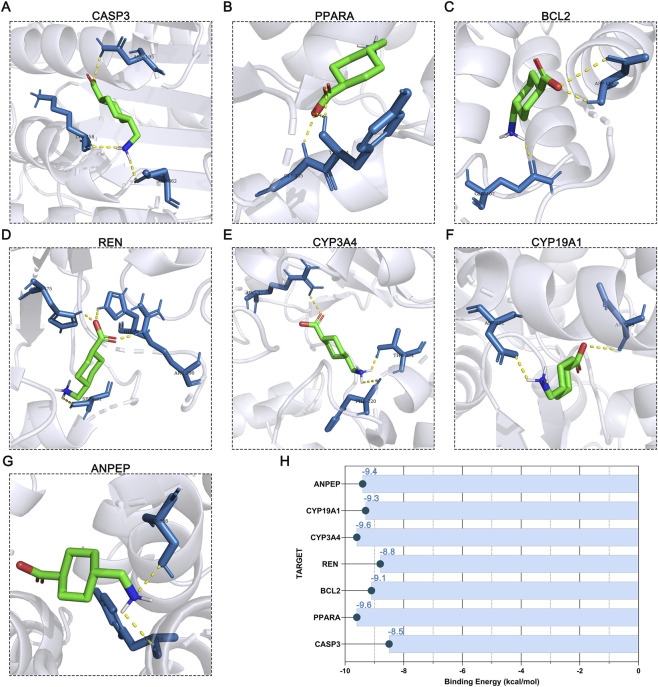
Molecular docking and scoring of key targets with TXA **(A)** Schematic diagram of TXA binding to CASP3 **(B)** Schematic diagram of TXA binding to PPARA **(C)** Schematic diagram of TXA binding to BCL2 **(D)** Schematic representation of TXA binding to REN **(E)** Schematic representation of TXA binding to CYP3A4 **(F)** Schematic representation of TXA binding to CYP19A1 **(G)** Schematic representation of TXA binding to ANPEP **(H)** Docking scores for TXA and key targets.

Molecular docking analyses were performed using the AutoDock platform to evaluate the binding interactions between TXA and the identified core target proteins. All docking simulations produced binding energies below −8.0 kcal/mol, indicating thermodynamically favorable interactions and supporting the biological relevance of the predicted targets (Figure 4). Visualization using PyMOL revealed the lowest-energy binding conformations, providing structural insight into the ligand–protein interaction modes and suggesting potential molecular mechanisms underlying TXA-associated pharmacological and toxicological effects in ICH (Supplementary Figure S4). Among the seven core targets, CASP3 exhibited the strongest binding affinity and the lowest docking energy with TXA (Table 3).

To further validate the docking results, virtual alanine-scanning mutagenesis was performed using Discovery Studio. Amino acid residues within a 3 Å radius of TXA in the ligand–protein complex were individually mutated to alanine (ALA). Mutations at ASP362, MET226, GLN107, ASP396, ARG106, and ASP222 resulted in increased binding energies (Δenergy > 0.5) and reduced interaction stability, indicating that these residues are likely critical for TXA binding (Supplementary Figure S5). These findings further support the reliability of the molecular docking predictions.

#### 4.4.5 MD simulation and binding stability assessment

**FIGURE 5:**
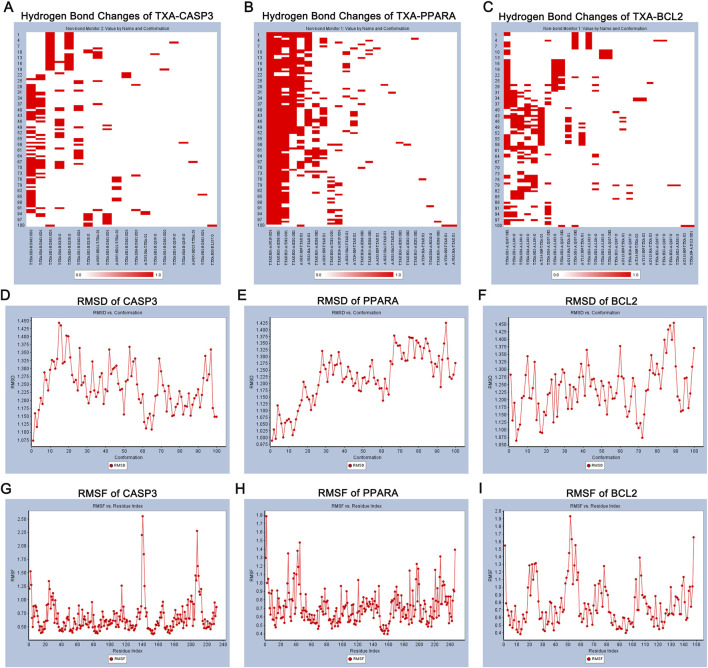
Molecular dynamics simulations of the TXA-key target complex **(A–C)** Molecular dynamics simulations of the TXA-key gene complex reveal time-dependent changes in binding hydrogen bonds **(D–F)** Molecular dynamics simulations of the TXA-key gene complex demonstrate protein stability within 100 nanoseconds (RMSD < 1.45 nm) **(G–I)** RMSF analysis reveals flexible protein residues and stable interactions with the ligand, further confirming the stability of the binding interaction between TXA and the key gene.

MD simulations were performed using Discovery Studio to evaluate the structural stability, flexibility, and compactness of the TXA–CASP3, TXA–PPARA, and TXA–BCL2 complexes. Key parameters, including RMSD, RMSF, and dynamic hydrogen bond formation, were systematically analyzed (Figure 5). During the 100 ns simulation, all three protein–ligand complexes maintained stable conformations. RMSD analysis showed that each complex reached equilibrium within approximately 20 ns and remained stable thereafter, with RMSD values consistently below 2.0 Å throughout the simulation. Dynamic hydrogen-bond analysis revealed continuous yet stable interaction patterns between TXA and the target proteins. RMSF analysis demonstrated that fluctuations of both backbone and side-chain residues remained within the 0–2.0 Å range, indicating limited local flexibility and overall structural stability of the complexes under simulated physiological conditions. Collectively, these results suggest that the TXA–target interactions represent thermodynamically stable binding states rather than transient associations, supporting their biological relevance and potential for sustained pharmacological effects.

### 4.5 Preliminary validation through *in vitro* experiments

#### 4.5.1 Effects of TXA exposure on hippocampal neurons

**FIGURE 6:**
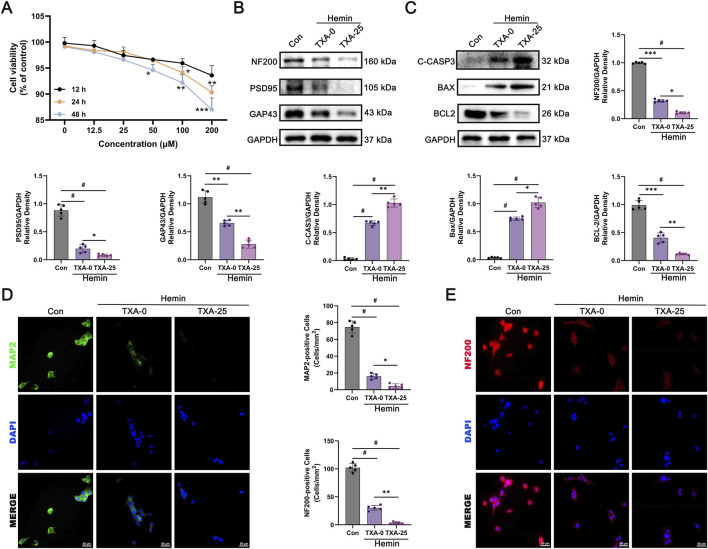
*In vitro* validation of TXA’s effects on ICH disease: Based on the HT22 hippocampal neuronal cell model **(A)** CCK8 assay screening for optimal TXA concentration and pretreatment duration **(B)** Western blot analysis of TXA’s effects on axon-related proteins in HT22 cells post-ICH **(C)** Western blot analysis of TXA’s effects on apoptosis-related proteins in HT22 cells post-ICH **(D)** Live/dead cell staining to detect the effect of TXA on MAP2 expression in HT22 cells after ICH. Scale bar = 50 μm **(E)** Immunofluorescence assay to detect the effect of TXA on NF200 expression in HT22 cells after ICH. Scale bar = 50 μm. All Western blot images are from independent replicate experiments and are representative of the results. All data are expressed as mean ± standard deviation (SD). Statistical significance was determined by two-way analysis of variance (ANOVA) and Tukey’s multiple comparison test: *p < 0.05, **p < 0.01, and ***p < 0.001 vs. TAX-0 group, n ≥ 3. ns: not significant.

An *in vitro* model was established to evaluate the effects of TXA exposure on hippocampal neurons under hemin-induced injury conditions. HT22 cells were treated with gradient concentrations of TXA for 12, 24, and 48 h, and cell viability was assessed using the CCK-8 assay. Hemin stimulation was used to mimic ICH-related injury, with 25 μM TXA administered for 12 h as a pretreatment condition (Figure 6A). WB analysis showed that HT22 cells pretreated with hemin and subsequently exposed to TXA exhibited further reductions in the expression of neuronal and axonal markers, including NF200, PSD95, and GAP43, compared with hemin treatment alone (Figure 6B). To investigate apoptosis-related changes, the expression of apoptosis-associated proteins was examined. In the hemin + TXA group, the levels of pro-apoptotic proteins CASP3 and Bax were significantly increased, whereas the anti-apoptotic protein Bcl-2 was markedly decreased (Figure 6C). In addition, C-CASP3 increases progressively as TXA concentrations rise, a trend that generally correlates with the decline in cell viability (Supplementary Figure S6). Consistent with these findings, immunofluorescence staining revealed reduced expression of the neuronal marker MAP2 and the axonal protein NF200 in HT22 cells treated with combined hemin and TXA (Figures 6D,E). Notably, TXA exposure was associated with decreases not only in fluorescence intensity but also in the number of MAP2- and NF200-positive cells. These results suggest that TXA exposure may exacerbate neuronal structural damage and apoptosis under hemin-induced injury conditions. Based on the results of molecular docking and molecular dynamics simulations, which strongly suggest that CASP3 is a likely binding target through which TXA influences HT22 cell survival, we conducted CETSA and DARTS experiments. The results showed that in the presence of TXA, the thermal stability of CASP3 was significantly increased (i.e., more undenatured protein remained after heating), as evidenced by a rightward shift in the melting curve. Concurrently, DARTS experiments indicated that the addition of TXA at various concentrations effectively slowed the degradation rate of CASP3. This provides preliminary evidence of a direct binding between TXA and CASP3 (Supplementary Figure S6).

#### 4.5.2 Effects of TXA exposure on microglia

**FIGURE 7:**
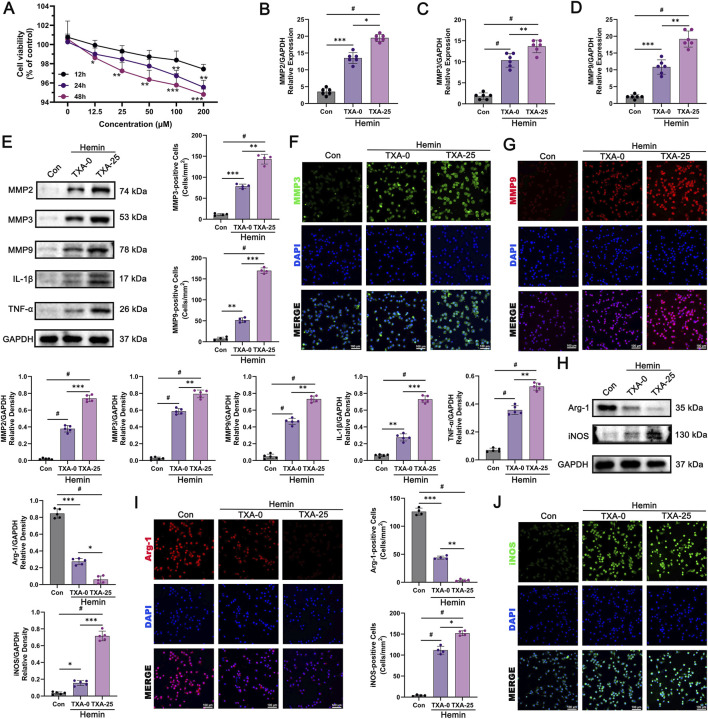
*In vitro* validation of TXA’s effects on ICH disease: Based on the BV2 glial cell model **(A)** CCK8 assay screening for optimal TXA concentration and pretreatment duration **(B–D)** q-PCR detection of MMP2/MMP3/MMP9 levels in BV2 cells across groups **(E)** Western blot analysis of TXA’s impact on MMPs and inflammatory factor expression in BV2 cells post-ICH **(F)** Immunofluorescence analysis of TXA’s effect on MMP3 expression in BV2 cells after ICH. Scale bar = 100 μm **(G)** Immunofluorescence analysis of TXA’s effect on MMP9 expression in BV2 cells after ICH. Scale bar = 100 μm **(H)** Western blot analysis of TXA’s effect on iNOS and Arg-1 protein expression in BV2 cells after ICH **(I)** Immunofluorescence analysis of the effect of TXA on Arg-1 expression in BV2 cells after ICH. Scale bar = 100 μm **(J)** Immunofluorescence analysis of the effect of TXA on iNOS expression in BV2 cells after ICH. Scale bar = 100 μm. All Western blot images are from independent replicate experiments and are representative of the results. All data are expressed as mean ± standard deviation (SD). Statistical significance was determined by two-way analysis of variance (ANOVA) and Tukey’s multiple comparison test: *p < 0.05, **p < 0.01, and ***p < 0.001 vs. TAX-0 μM group, n ≥ 3. ns: not significant.

Given the pivotal role of microglia in ICH pathophysiology, BV2 microglial cells were used for *in vitro* validation. BV2 cells were treated with gradient concentrations of TXA for 12, 24, and 48 h, and cell viability was assessed using the CCK-8 assay. Treatment with 25 μM TXA for 24 h was selected for subsequent experiments (Figure 7A). Quantitative real-time PCR analysis showed that TXA exposure significantly increased the mRNA expression levels of MMP2, MMP3, and MMP9 in hemin-treated BV2 cells (Figures 7B–D). WB analysis demonstrated elevated MMP protein levels, accompanied by increased expression of the pro-inflammatory cytokines IL-1β and TNF-α following TXA treatment (Figure 7E). Immunofluorescence staining further confirmed increased expression of MMP3 and MMP9 in BV2 cells subjected to combined hemin and TXA treatment, in agreement with the qPCR and WB results (Figures 7F,G). To evaluate the effect of TXA on microglial polarization, polarization-associated markers were analyzed by WB. TXA treatment increased iNOS expression and decreased Arg-1 expression in hemin-treated BV2 cells (Figure 7H). Immunofluorescence analysis showed corresponding changes in iNOS and Arg-1 fluorescence intensity (Figures 7I,J), supporting the protein expression results. Collectively, these findings indicate that TXA exposure enhances matrix metalloproteinase expression and promotes a pro-inflammatory phenotype in microglia under hemin-induced injury conditions.

## 5 Discussion

In recent decades, the global incidence of hemorrhagic stroke has increased substantially, remaining associated with high mortality and disability rates and limited therapeutic options. Considerable efforts have been devoted to identifying effective interventions to mitigate the heavy burden imposed by ICH on patients and society. Although clinical research investigating TXA in ICH has expanded, its potential neurotoxicity has received comparatively limited attention. The present study demonstrates the value of integrating pharmacovigilance data mining, network toxicology analysis, and experimental validation to comprehensively evaluate TXA safety in cerebrovascular disease contexts.

Demographic analysis of the FAERS database showed that females accounted for the majority of reported AEs, consistent with known pharmacoepidemiological patterns and previous reports of higher AE reporting rates among women. This observation may reflect differences in reporting behavior or greater exposure to TXA in female-specific clinical settings, such as postpartum hemorrhage (Kim et al., 2024). Age-stratified analysis suggested a lower reporting frequency among older individuals, which may be attributable to reporting bias, altered drug tolerance, or underreporting in elderly populations; however, this finding requires confirmation in prospective studies. Importantly, most reports were submitted by healthcare professionals, supporting the reliability and clinical relevance of the adverse event reporting data. The predominance of reports originating from the United States highlights the inherent geographic bias of the FAERS database and underscores the need for validation in other populations and healthcare systems. At the PT level, strong safety signals were observed for seizure-related events, dystonic tremor, and thrombotic complications, suggesting an association between TXA exposure and neurological adverse outcomes. These findings are supported by experimental evidence from animal studies demonstrating that TXA can exert pro-epileptic effects through direct action on the central nervous system (Serdoğan et al., 2025; Irl et al., 2017). Previous studies have shown that intracortical or intracerebroventricular administration of TXA can induce generalized seizures, indicating that elevated intracerebral concentrations may increase seizure susceptibility (Schlag et al., 2002). Time-series analysis revealed a peak in TXA-related AE reports in 2019, followed by a gradual decline, consistent with a typical Weber effect observed in post-marketing surveillance. This pattern may be associated with increased clinical utilization and heightened awareness of TXA during that period (Ahma et al., 2020). Accurate identification of such temporal patterns is valuable for interpreting drug safety signals and preventing misjudgments in monitoring.

Network toxicology analysis revealed potential molecular mechanisms underlying TXA-associated adverse effects in ICH, linking computational predictions to biological interpretation. CASP3, PPARA, and BCL2 were identified as core targets that may mediate both the therapeutic and adverse effects of TXA, offering new insights into its molecular actions in ICH. Molecular docking demonstrated strong binding affinities between TXA and these targets, with binding energies below −8.0 kcal/mol, supporting the biological relevance of the predicted interactions. Among these targets, CASP3 occupied a central position within the adverse effect network, suggesting that pathways contributing to therapeutic efficacy may also underlie tolerability concerns. MD simulations further showed that TXA–target complexes remained stable over a 100 ns simulation period, with RMSD values consistently within 2.0 nm and limited RMSF fluctuations at binding sites, indicating stable and specific interactions. Collectively, these findings support the potential biological significance of the identified TXA–gene interactions at the molecular level (Forli et al., 2016). Nevertheless, it should be emphasized that molecular docking and dynamics simulations are inherently predictive and exploratory. The proposed binding modes and interaction patterns require further validation through biochemical techniques, such as surface plasmon resonance, as well as functional validation in cellular and *in vivo* models. Thus, the present results should be interpreted as hypothesis-generating rather than definitive mechanistic evidence.

*In vitro* experiments were performed using HT22 hippocampal neurons and BV2 microglial cells, with key gene and protein expression assessed by quantitative real-time PCR, WB, and immunofluorescence. In HT22 cells, TXA exposure under hemin-induced injury conditions impaired cell viability and reduced the expression of neurofilament and axonal proteins, indicating aggravated neuronal structural damage. In BV2 cells, TXA treatment markedly enhanced microglial inflammatory activation, as evidenced by increased expression of MMPs, IL-1β, and TNF-α. Moreover, analysis of polarization markers demonstrated that TXA accelerated the shift of hemin-treated BV2 cells toward a pro-inflammatory phenotype. Collectively, these findings suggest that TXA exposure may exacerbate neuroinflammatory responses following ICH by simultaneously affecting neuronal integrity and microglial activation. However, it should be noted that these observations were derived from a single *in vitro* cellular model and may not fully recapitulate the complex pathophysiological environment of human ICH. Further validation in animal models and clinical settings is required.

Nevertheless, several limitations should be considered when interpreting these findings. A principal limitation is that the primary pharmacovigilance analysis was not restricted to ICH-diagnosed patients. The reported ADE signals reflect TXA’s safety profile across all clinical uses. Therefore, the extrapolation of these findings to the specific context of ICH is preliminary and requires confirmation in dedicated ICH patient cohorts. Our subsequent ICH-focused analyses (network toxicology and *in vitro* experiments) are hypothesis-generating and should be interpreted in light of this limitation. Moreover, Several methodological strengths and limitations related to our sensitivity and stratified analyses should be noted. The consistency of the primary neurological signals across sensitivity analyses (e.g., restriction to serious reports, healthcare professional reports) supports the robustness of these findings. However, the sample size in some subgroups (e.g., specific age-sex strata) was limited, which may have reduced statistical power to detect signals for rarer adverse events. Furthermore, despite our stratification efforts, residual confounding by indication, co-medication, and underlying disease severity cannot be fully excluded, as the FAERS database does not capture detailed clinical information. The observed sex and age differences should be interpreted as hypothesis-generating rather than definitive, given the inherent reporting biases in spontaneous reporting systems. Future prospective studies with standardized data collection are needed to confirm these subgroup findings. In addition, the molecular docking and molecular dynamics simulations presented in this study (Section 4.34–4.35) predicted stable binding between TXA and core targets, including CASP3, PPARA, and BCL2, with binding energies below −8.0 kcal/mol and RMSD values below 2.0 Å. However, these computational predictions remain hypothetical until validated by direct biochemical assays. Several approaches could provide more conclusive evidence in future studies: (1) Surface plasmon resonance (SPR) or isothermal titration calorimetry (ITC) to directly measure binding affinity (KD) and thermodynamic parameters between purified recombinant proteins and TXA; (2) Cellular thermal shift assay (CETSA) to confirm target engagement of TXA with endogenous PPARA, or BCL2 in living cells; (3) Site-directed mutagenesis of the predicted key residues (e.g., ASP362, MET226, GLN107 of CASP3, as identified in Supplementary Figure S5) followed by binding assays to validate the predicted interaction hotspots. Second, although network toxicology, molecular docking, and MD simulations offer valuable mechanistic hypotheses, these computational predictions require further validation through rigorous *in vitro* and *in vivo* experiments. Future studies employing animal models of ICH are necessary to validate the neurotoxic and pro-inflammatory effects of TXA. Such *in vivo* experiments would enable assessment of clinically relevant endpoints, including hematoma volume dynamics, neurological deficit scores, brain edema, and long-term functional recovery, as well as immunohistochemical evaluation of neuronal death, microglial polarization, and expression of CASP3, PPARA, and BCL2 in perihematomal tissues.

Several intrinsic biases associated with spontaneous reporting systems, such as the FAERS database, warrant careful consideration when interpreting the present findings. Indication bias. Tranexamic acid is primarily used in clinical settings with inherent thrombotic or neurological risks, including trauma, major surgery, postpartum hemorrhage, and cardiac procedures. Patients receiving TXA may therefore have a higher baseline risk for adverse events such as thromboembolism or seizures, independent of drug exposure. This indication bias could lead to an overestimation of the true association between TXA and certain adverse events. For example, the strong signal for cerebral artery thrombosis may partly reflect the underlying prothrombotic state of the bleeding condition rather than a direct drug effect. Our study could not fully adjust for indication because the FAERS database does not systematically capture detailed clinical indications. Reporting bias. Spontaneous reporting is subject to multiple forms of reporting bias. The Weber effect—a temporal pattern characterized by a peak in reporting during the first few years after market introduction followed by a gradual decline—was observed in our time-series analysis (Supplementary Figure S2). This pattern does not necessarily reflect a true change in drug safety but rather fluctuations in clinical awareness and media attention. Additionally, “alarm bias” may occur when a serious adverse event is first reported, leading to a transient surge in subsequent reports of the same event. The predominance of reports from the United States and from healthcare professionals further indicates geographical and professional reporting disparities, limiting the generalizability of our findings to other regions or patient populations. Confounding by co-medication. Confounding by co-medication is particularly relevant in the perioperative setting, where TXA is frequently administered. Many drugs commonly used during surgery have known proconvulsant properties. The FAERS database does not systematically capture these concomitant drugs or their dosing regimens. Therefore, some of the seizure signals attributed to TXA in our analysis could be partially or fully confounded by concurrent perioperative medications. This limitation is particularly relevant for signals such as ‘myoclonic seizure’ and ‘status epilepticus’. Future pharmacovigilance studies using electronic health records or large-scale registry data that include complete medication lists are needed to disentangle the independent effect of TXA from that of co-administered agents. The FAERS database does not routinely provide sufficient detail on concomitant medications, dosing, or timing to allow adequate control for such confounding. Therefore, the signals detected in this study should be interpreted as associations rather than causal relationships. Missing data and incomplete reporting. As noted in the demographic analysis (Section 4.1.1), a substantial proportion of reports lacked information on key variables, including sex, age, and route of administration. Missing data can introduce bias if the absence of information is not random. Our approach of creating an “Missing” category for missing values does not eliminate this bias but rather makes its extent transparent. Future pharmacovigilance studies using more structured data sources, such as electronic health records or registries, are needed to confirm the findings presented here. Despite these limitations, the triangulation of pharmacovigilance signals with network toxicology predictions and *in vitro* experimental data strengthens the overall evidence for TXA-associated neurotoxicity. The consistency of the primary neurological signals across multiple sensitivity and stratified analyses (Section 4.3) also supports the robustness of our findings against certain types of bias.

Finally, although the present *in vitro* experiments primarily used a single TXA concentration (25 μM) for mechanistic readouts, our CCK-8 viability data clearly demonstrate a concentration-dependent effect of TXA on both HT22 neurons and BV2 microglia under hemin-induced injury. The IC_50_ values (∼32–38 μM) are within or slightly above the peak plasma concentrations achieved after standard intravenous TXA administration (10–30 μM for a 1 g bolus). This suggests that the neurotoxic and pro-inflammatory effects observed at 25 μM are clinically relevant. However, we acknowledge that we did not perform full dose-response analyses for all molecular endpoints (e.g., CASP3, MMP9, iNOS). It remains possible that different molecular markers exhibit different concentration thresholds. For instance, microglial MMP9 expression might increase at lower TXA concentrations than neuronal apoptosis markers, which would have implications for the therapeutic window of TXA in ICH. Future studies systematically mapping concentration–effect curves for each key target are warranted. Until such data are available, our findings should be interpreted as evidence of toxicity at a fixed, clinically relevant concentration, with the understanding that the true dose–response relationship is likely nonlinear and may vary across cell types and endpoints.

Overall, this study presents a comprehensive evaluation framework integrating pharmacovigilance data mining, computational toxicology, and preliminary experimental validation, thereby strengthening the assessment of TXA safety in ICH. By combining traditional drug-safety analysis with advanced computational approaches, this work provides new insights into the potential neurotoxic and pro-inflammatory effects of TXA in ICH and proposes a scalable methodological strategy to address broader pharmacovigilance challenges. Importantly, translating these findings into clinically actionable risk assessment tools may improve therapeutic decision-making and advance the goals of precision pharmacovigilance and personalized medicine.

## 6 Conclusion

**FIGURE 8:**
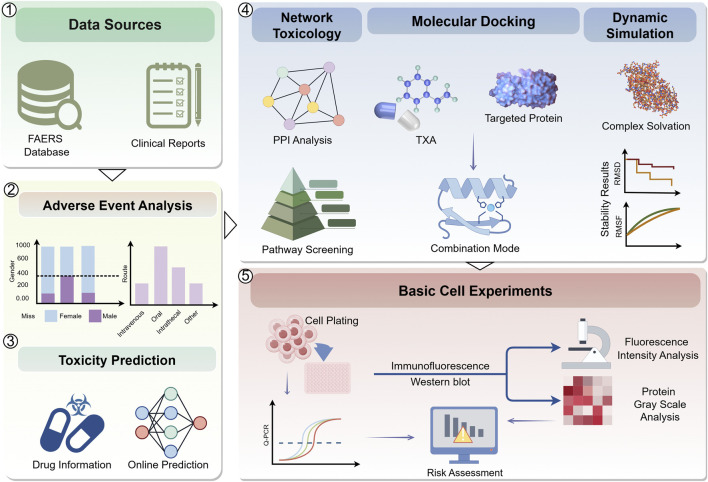
This diagram illustrates the computational and validation framework for enhanced pharmacovigilance, demonstrating the systematic integration of data sources, toxicity prediction, toxicological analysis, and experimental modules. It aims to achieve comprehensive safety monitoring and regulation of TXA within the ICH framework.

TXA is widely used for the management of intraoperative bleeding, reduction of allogeneic transfusion, and postpartum hemorrhage. In this study, we systematically evaluated the potential neurotoxicity of TXA in ICH by integrating pharmacovigilance analysis of the FAERS database, network toxicology, molecular docking and MD simulations, and *in vitro* experimental validation (Figure 8). FAERS analysis revealed a progressive increase in TXA-related adverse event reports, with a notable peak in 2019, and identified multiple significant nervous system–related adverse drug event signals, including high-signal events such as myoclonic seizures and status epilepticus. Network toxicology analysis identified 58 TXA-associated targets related to ICH, from which seven core targets (CASP3, PPARA, BCL2, REN, CYP3A4, CYP19A1, and ANPEP) were highlighted through protein–protein interaction analysis and computational validation. Molecular docking and dynamics simulations revealed stable, biologically relevant interactions between TXA and these targets. *In vitro* experiments further demonstrated that TXA exposure aggravated neuronal structural damage and reduced cell viability in hemin-treated HT22 cells, while promoting inflammatory activation and pro-inflammatory polarization in hemin-treated BV2 microglia. Collectively, these findings suggest that TXA may exert neurotoxic and pro-inflammatory effects that could adversely influence ICH outcomes. To our knowledge, this study provides the first comprehensive evidence linking TXA exposure to potential neurotoxicity in the context of ICH. These results underscore the importance of prioritizing safety evaluation of TXA in neurological conditions and highlight the need for cautious clinical application and close monitoring of adverse neurological events in future ICH-related studies and trials.

## Data Availability

The datasets presented in this study can be found in online repositories. The names of the repository/repositories and accession number(s) can be found below: Mendeley Data, V1, doi: 10.17632/kzj2mnfgwh.1.
