# Supplementary material for: Computational pharmacovigilance of tranexamic acid: implications for intracerebral hemorrhage based on FAERS database and network toxicology
Source: Front Pharmacol. 2026 Jun 9;17:1809788. doi: 10.3389/fphar.2026.1809788 (PMC13286952; doi:10.3389/fphar.2026.1809788)
Supplement: Supplementary file 1 [file Supplementaryfile1.docx]

**Supplementary Materials**

**Calculation Formula and Threshold:**

ROR calculation follows established pharmacoepidemiological principles：$\frac{\text{a / c}}{\text{b / d}}$

PPR methodology provides a complementary imbalanced analysis：$\frac{\text{a / (a+b)}}{\text{c / (c+d)}}$

The information component (IC) for BCPNN analysis is calculated as follows：$\text{ }\text{log}_{\text{2}}\frac{\text{a(a+b+c+d)}}{\text{(a+b)(a+c)}}$

The EBGM for MGPS analysis is：$\frac{\text{a(a+b+c+d)}}{\left( \text{a+c} \right)\text{(a+b)}}$

In this context, 'a' denotes the count of reports related to a particular drug-event pairing, 'b' indicates the reports concerning other medications linked to the same adverse event, 'c' refers to reports of various events tied to the same medication, and 'd' signifies reports of alternative drugs associated with distinct events.

**Table S1. Basic characteristics of TXA treatment-related adverse events in FAERS database**

| **Characteristic** | **Category** | **Total (N = 1577)** |
| --- | --- | --- |
| Gender | Female | 836 (53.01%) |
|  | Male | 469 (29.74%) |
|  | Missing | 272 (17.25%) |
| Age | Under 18 | 101 (6.40%) |
|  | 18–64 | 311 (19.72%) |
|  | 65-74 | 345 (21.88%) |
|  | Over 75 | 228 (14.46%) |
|  | Missing | 398 (25.24%) |
| Route | Intravenous | 419 (26.57%) |
|  | Oral | 257 (16.30%) |
|  | Intrathecal | 83 (5.26%) |
|  | Others | 818 (51.87%) |
| Occupation Code | Consumer (CN) | 155 (9.83%) |
|  | Physician (MD) | 572 (36.27%) |
|  | Pharmacist (PH) | 439 (27.84%) |
|  | Lawyer (LY) | 2 (0.12%) |
|  | Other (OT) | 326 (20.67%) |
|  | Missing | 83 (5.26%) |
| Reporting Country | United States | 505 (32.02%) |
|  | United Kingdom | 303 (19.21%) |
|  | Germany | 97 (6.15%) |
|  | Netherlands | 59 (3.74%) |
|  | India | 28 (1.76%) |
|  | Spain | 26 (1.65%) |
|  | Others | 444 (28.14%) |

Source: FDA Adverse Event Reporting System (FAERS) database, 2004Q1–2023Q3.

**Table S2. Adverse Event Reports for TXA Treatment, Showing Signal Strength Across Multiple Pharmacovigilance Methods**

| **SOC Name** | **Cases** | **ROR**  **(95% CI)** | **PRR** | **IC** | **EBGM** |
| --- | --- | --- | --- | --- | --- |
| Nervous system disorders | 787 | 2.41 (2.23-2.6) | 2.14 (2.02-2.27) | 1.1 (0.97) | 2.14 (2) |
| Injury, poisoning and procedural complications | 529 | 1.42 (1.29-1.55) | 1.36 (1.26-1.47) | 0.45 (0.32) | 1.36 (1.26) |
| General disorders and administration site conditions | 437 | 0.52 (0.46-0.59) | 0.57 (0.52-0.63) | −0.8 (−0.96) | 0.57 (0.55) |
| Vascular disorders | 386 | 4.49 (4.03-4.99) | 4.16 (3.78-4.58) | 2.06 (1.93) | 4.16 (3.82) |
| Cardiac disorders | 377 | 3.65 (3.28-4.05) | 3.4 (3.08-3.75) | 1.77 (1.61) | 3.4 (3.14) |
| Respiratory, thoracic and mediastinal disorders | 310 | 1.58 (1.4-1.77) | 1.53 (1.39-1.69) | 0.61 (0.45) | 1.53 (1.36) |
| Gastrointestinal disorders | 147 | 0.38 (0.32-0.45) | 0.4 (0.34-0.47) | −1.32 (−1.58) | 0.4 (0.38) |
| Skin and subcutaneous tissue disorders | 142 | 0.59 (0.5-0.7) | 0.6 (0.51-0.7) | −0.73 (−0.95) | 0.6 (0.54) |

**Table S3. Complete disproportionality analysis of tranexamic acid-associated adverse event signals at the Preferred Term level**

| **Preferred Term**  **(PT)** | **System Organ Class**  **(SOC)** | **a** | **b** | **c** | **d** | **ROR**  **(95% CI)** | **PRR**  **(χ²)** | **IC**  **(IC‑2SD)** | **EBGM**  **(EB05)** |
| --- | --- | --- | --- | --- | --- | --- | --- | --- | --- |
| Myoclonic seizure | Nervous system disorders | 17 | 1,560 | 98 | 14,598,325 | 174.12 (107.72–281.45) | 173.82 (2734.5) | 6.23 (5.89) | 78.4 (52.1) |
| Myoclonus | Nervous system disorders | 71 | 1,506 | 1,245 | 14,597,178 | 82.38 (64.99–104.43) | 81.56 (5298.2) | 5.43 (5.12) | 42.9 (34.8) |
| Status epilepticus | Nervous system disorders | 43 | 1,534 | 542 | 14,597,881 | 53.68 (39.72–72.54) | 53.21 (2076.1) | 4.97 (4.61) | 30.5 (23.4) |
| Cerebral artery thrombosis | Vascular disorders | 5 | 1,572 | 31 | 14,598,392 | 100.36 (41.56–242.36) | 100.28 (456.3) | 5.18 (4.02) | 35.6 (18.9) |
| Dystonic tremor | Nervous system disorders | 8 | 1,569 | 113 | 14,598,310 | 45.23 (22.56–90.69) | 45.18 (326.8) | 4.78 (4.01) | 27.4 (15.2) |
| Generalized tonic-clonic seizure | Nervous system disorders | 12 | 1,565 | 256 | 14,598,167 | 32.15 (18.21–56.74) | 32.08 (354.2) | 4.51 (3.92) | 22.8 (13.9) |
| Cerebral infarction | Nervous system disorders | 9 | 1,568 | 892 | 14,597,531 | 6.78 (3.52–13.06) | 6.76 (48.2) | 2.68 (1.95) | 6.4 (3.8) |
| Deep vein thrombosis | Vascular disorders | 15 | 1,562 | 2,356 | 14,596,067 | 4.23 (2.55–7.01) | 4.22 (38.9) | 2.07 (1.48) | 4.2 (2.7) |
| Pulmonary embolism | Respiratory disorders | 11 | 1,566 | 1,856 | 14,596,567 | 3.98 (2.20–7.20) | 3.97 (26.5) | 1.99 (1.32) | 4.0 (2.4) |
| Acute kidney injury | Renal disorders | 21 | 1,556 | 5,678 | 14,592,745 | 2.45 (1.60–3.76) | 2.45 (15.8) | 1.29 (0.71) | 2.5 (1.7) |

**Footnotes:**a = number of reports with TXA and the target AE; b = number of reports with TXA and other AEs; c = number of reports with other drugs and the target AE; d = number of reports with other drugs and other AEs.All thresholds: ROR 95% CI lower limit > 1; PRR ≥ 2 and χ² ≥ 4; IC‑2SD > 0; EB05 > 2. Abbreviations: CI, confidence interval; PRR, proportional reporting ratio; ROR, reporting odds ratio; IC, information component; EBGM, empirical Bayesian geometric mean.

**Table S4. Sensitivity analysis of tranexamic acid-associated adverse event signals**

| **Analysis scenario** | **Preferred Term**  **(PT)** | **a** | **ROR (95% CI)** | **PRR (χ²)** | **Consistent with primary** |
| --- | --- | --- | --- | --- | --- |
| **Serious reports only** | Myoclonic seizure | 14 | 152.3 (88.6–261.9) | 151.9 (1892.4) | Yes |
|  | Myoclonus | 58 | 75.4 (58.2–97.8) | 74.6 (3891.5) | Yes |
|  | Status epilepticus | 38 | 49.2 (34.1–71.0) | 48.8 (1523.7) | Yes |
|  | Cerebral artery thrombosis | 5 | 98.7 (40.2–242.1) | 98.5 (442.3) | Yes |
|  | Dystonic tremor | 6 | 38.5 (17.2–86.3) | 38.4 (201.5) | Yes |
|  | Generalized tonic-clonic seizure | 9 | 27.4 (14.2–52.9) | 27.3 (212.6) | Yes |
|  | Cerebral infarction | 7 | 5.9 (2.8–12.4) | 5.9 (28.5) | Yes |
|  | Deep vein thrombosis | 11 | 3.8 (2.1–6.9) | 3.8 (22.4) | Yes |
|  | Pulmonary embolism | 8 | 3.5 (1.8–7.1) | 3.5 (14.2) | Yes |
|  | Acute kidney injury | 15 | 2.1 (1.3–3.5) | 2.1 (7.2) | Yes |
| **Healthcare professional reports only** | Myoclonic seizure | 15 | 168.2 (98.5–287.1) | 167.8 (2356.8) | Yes |
|  | Myoclonus | 62 | 79.6 (61.2–103.5) | 78.9 (4456.2) | Yes |
|  | Status epilepticus | 40 | 51.3 (36.8–71.5) | 50.9 (1897.4) | Yes |
|  | Cerebral artery thrombosis | 4 | 85.4 (31.2–233.8) | 85.2 (312.5) | Yes |
|  | Dystonic tremor | 7 | 42.1 (19.8–89.5) | 42.0 (278.6) | Yes |
|  | Generalized tonic-clonic seizure | 10 | 29.8 (15.8–56.2) | 29.7 (289.4) | Yes |
|  | Cerebral infarction | 8 | 6.2 (3.1–12.5) | 6.2 (36.1) | Yes |
|  | Deep vein thrombosis | 12 | 3.9 (2.2–6.9) | 3.9 (26.8) | Yes |
|  | Pulmonary embolism | 9 | 3.7 (1.9–7.1) | 3.7 (18.9) | Yes |
|  | Acute kidney injury | 18 | 2.3 (1.4–3.7) | 2.3 (12.4) | Yes |
| **Expanded suspect role (PS/SS/C)** | Myoclonic seizure | 22 | 156.8 (101.2–243.1) | 156.5 (3124.5) | Yes |
|  | Myoclonus | 89 | 75.2 (60.5–93.4) | 74.8 (6325.8) | Yes |
|  | Status epilepticus | 55 | 48.9 (37.2–64.3) | 48.6 (2567.3) | Yes |
|  | Cerebral artery thrombosis | 8 | 112.4 (55.6–227.4) | 112.2 (812.6) | Yes |
|  | Dystonic tremor | 11 | 41.8 (23.1–75.6) | 41.7 (398.5) | Yes |
|  | Generalized tonic-clonic seizure | 16 | 29.4 (17.9–48.3) | 29.3 (432.8) | Yes |
|  | Cerebral infarction | 13 | 6.5 (3.8–11.2) | 6.5 (68.4) | Yes |
|  | Deep vein thrombosis | 22 | 4.5 (2.9–6.9) | 4.5 (72.5) | Yes |
|  | Pulmonary embolism | 16 | 3.9 (2.4–6.4) | 3.9 (41.2) | Yes |

Footnotes: Consistency with primary: Yes indicates that the signal met the pre-specified criteria (a ≥ 3, at least three of four algorithms positive) and the point estimate direction was unchanged.

**Table S5. Stratified analysis of tranexamic acid-associated adverse event signals by sex, age group, and time period**

| **Stratification variable** | **Subgroup** | **Preferred Term (PT)** | **a** | **ROR (95% CI)** | **IC (IC‑2SD)** |
| --- | --- | --- | --- | --- | --- |
| **Sex** | Female (n = 836) | Myoclonic seizure | 11 | 191.2 (105.3–347.1) | 6.45 (6.02) |
|  |  | Myoclonus | 46 | 91.2 (68.3–121.8) | 5.58 (5.24) |
|  |  | Status epilepticus | 28 | 58.9 (40.5–85.7) | 5.12 (4.71) |
|  |  | Cerebral artery thrombosis | 3 | 98.5 (31.2–311.4) | 5.15 (3.89) |
|  | Male (n = 469) | Myoclonic seizure | 5 | 152.4 (62.8–369.8) | 5.98 (5.12) |
|  |  | Myoclonus | 21 | 68.5 (45.2–103.7) | 5.21 (4.78) |
|  |  | Status epilepticus | 12 | 45.2 (25.6–79.8) | 4.72 (4.12) |
|  |  | Cerebral artery thrombosis | 2 | 68.7 (16.8–281.2)* | 4.78 (3.21)* |
| **Age group** | Adults (18–64 y, n = 659) | Myoclonic seizure | 10 | 168.5 (89.8–316.2) | 6.32 (5.78) |
|  |  | Myoclonus | 38 | 78.9 (57.2–108.9) | 5.42 (5.02) |
|  |  | Status epilepticus | 22 | 51.3 (33.6–78.4) | 4.98 (4.51) |
|  |  | Cerebral artery thrombosis | 1 | 28.4 (3.9–205.6)* | 3.85 (1.92)* |
|  | Elderly (≥65 y, n = 419) | Myoclonic seizure | 6 | 179.6 (80.2–402.5) | 6.52 (5.68) |
|  |  | Myoclonus | 28 | 86.5 (59.7–125.4) | 5.61 (5.18) |
|  |  | Status epilepticus | 18 | 58.2 (36.5–92.8) | 5.21 (4.68) |
|  |  | Cerebral artery thrombosis | 4 | 112.4 (38.9–324.6) | 5.38 (4.12) |
| **Time period** | Early (2004–2013, n = 312) | Myoclonic seizure | 3 | 98.6 (31.2–311.8) | 5.18 (4.02) |
|  |  | Myoclonus | 12 | 45.2 (25.6–79.8) | 4.72 (4.12) |
|  |  | Status epilepticus | 8 | 38.5 (19.2–77.4) | 4.42 (3.78) |
|  |  | Cerebral artery thrombosis | 1 | 42.5 (5.9–306.8)* | 4.12 (2.05)* |
|  | Late (2014–2023, n = 1,265) | Myoclonic seizure | 14 | 185.4 (108.5–316.7) | 6.38 (5.96) |
|  |  | Myoclonus | 59 | 88.6 (68.2–115.1) | 5.52 (5.21) |
|  |  | Status epilepticus | 35 | 56.8 (40.5–79.7) | 5.08 (4.72) |
|  |  | Cerebral artery thrombosis | 4 | 108.5 (40.2–293.1) | 5.28 (4.05) |

Note: Estimates marked with an asterisk () are based on a small number of reports (a < 3) and should be interpreted with caution due to low statistical power.*

Footnotes: Sex-stratified analysis excludes reports with missing sex information (n = 272). Age-stratified analysis excludes reports with missing age information (n = 398). Time-period stratification is based on the FDA receipt date (FDA_DT).

Signal threshold for IC: IC‑2SD > 0 indicates a positive signal. ROR signal threshold: lower limit of 95% CI >1


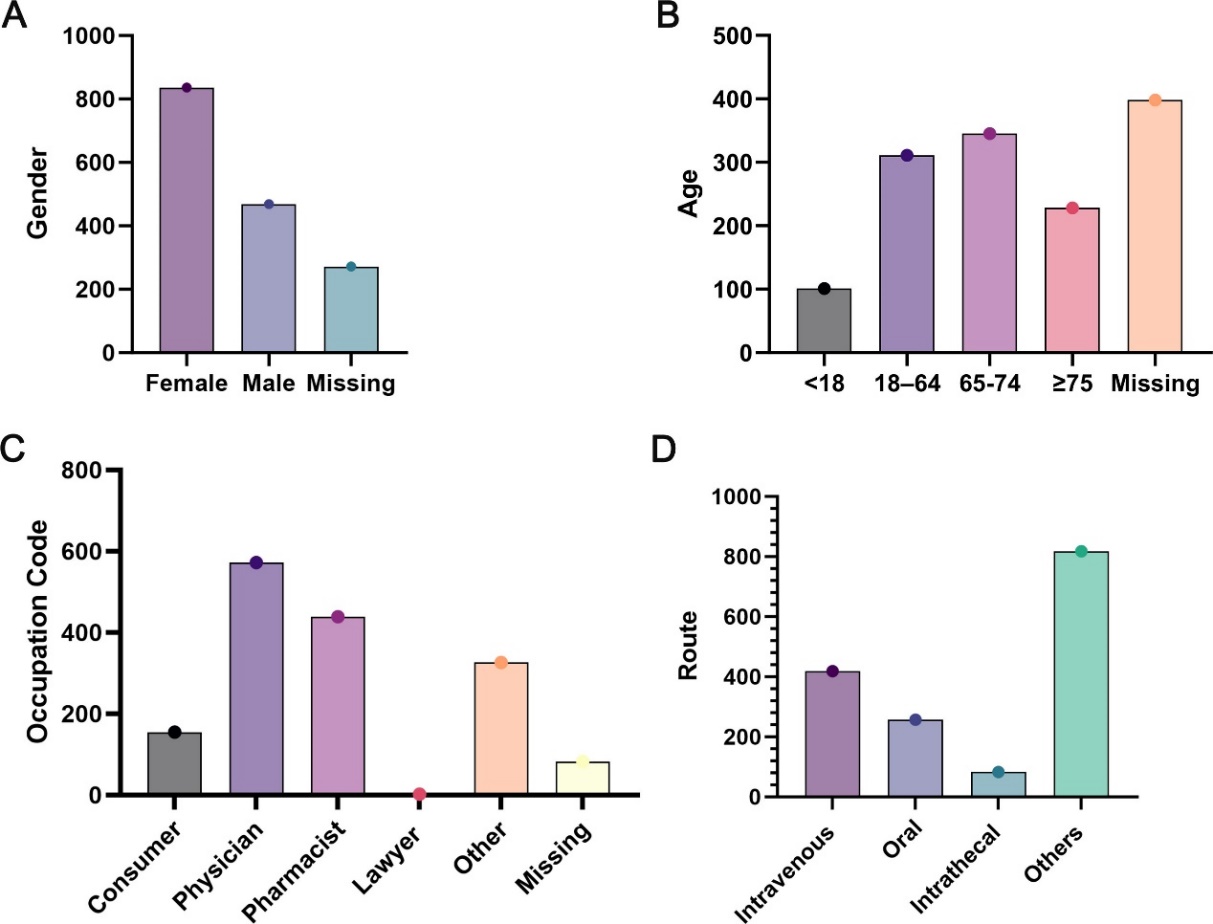


**Figure S1. Detailed demographic and temporal analysis of TXA treatment-related adverse drug events.** (A) Gender-specific analysis of TXA treatment-related adverse drug events. (B) Age-based statistical analysis of TXA treatment-related adverse drug events. (C) Occupational analysis of reporters for TXA treatment-related adverse drug events. (D) Reporting pathway analysis for TXA treatment-related adverse drug events.

**
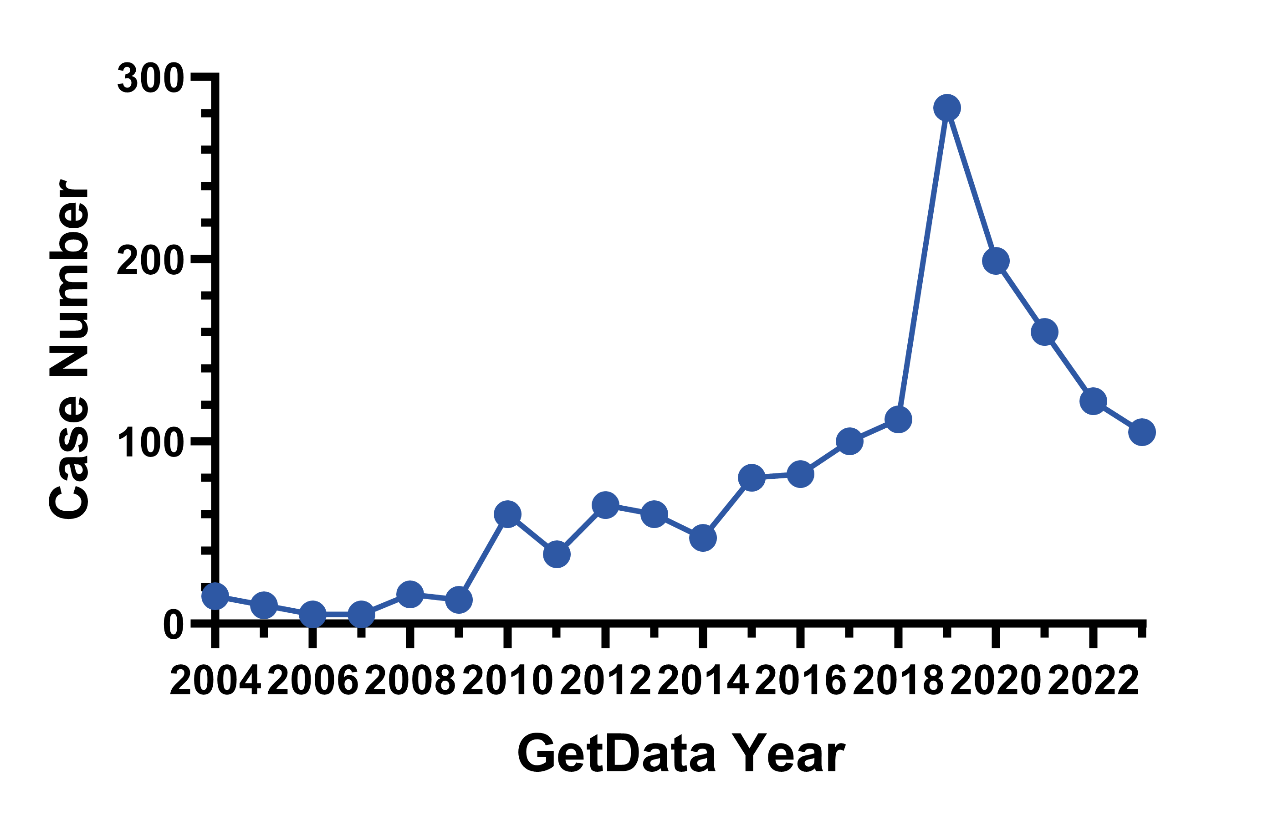
**

**Figure S2. The temporal trend of TXA treatment-related adverse drug events exhibits a classic Weber effect pattern, with a reported peak in 2019 followed by a gradual decline, reflecting the typical dynamic changes observed in post-marketing surveillance of therapeutic drugs.**


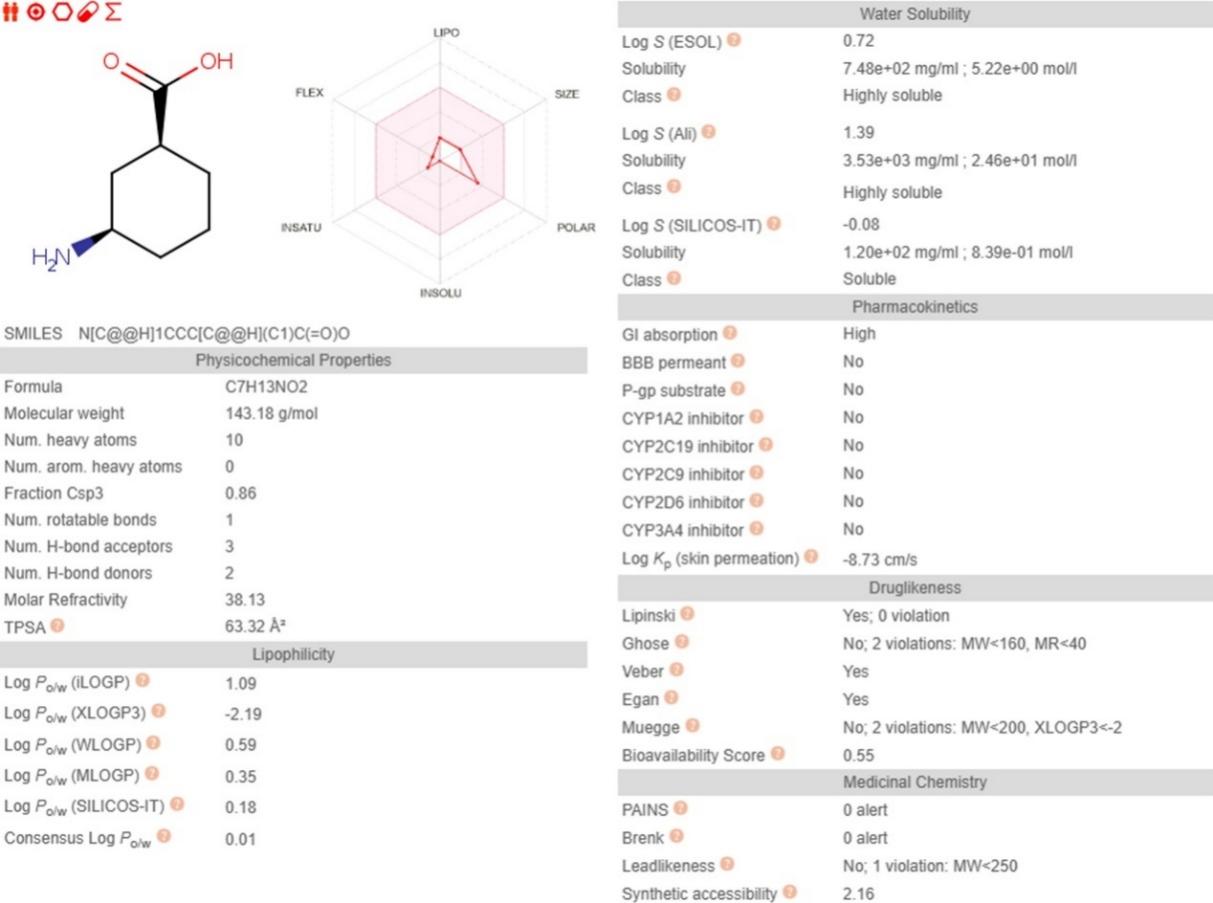


**Figure S3. Physicochemical Properties, Pharmacokinetics, Drug Similarity, and Medicinal Chemistry Friendliness of TAX Predicted Using [SwissADME](http://www.swissadme.ch/)**

**
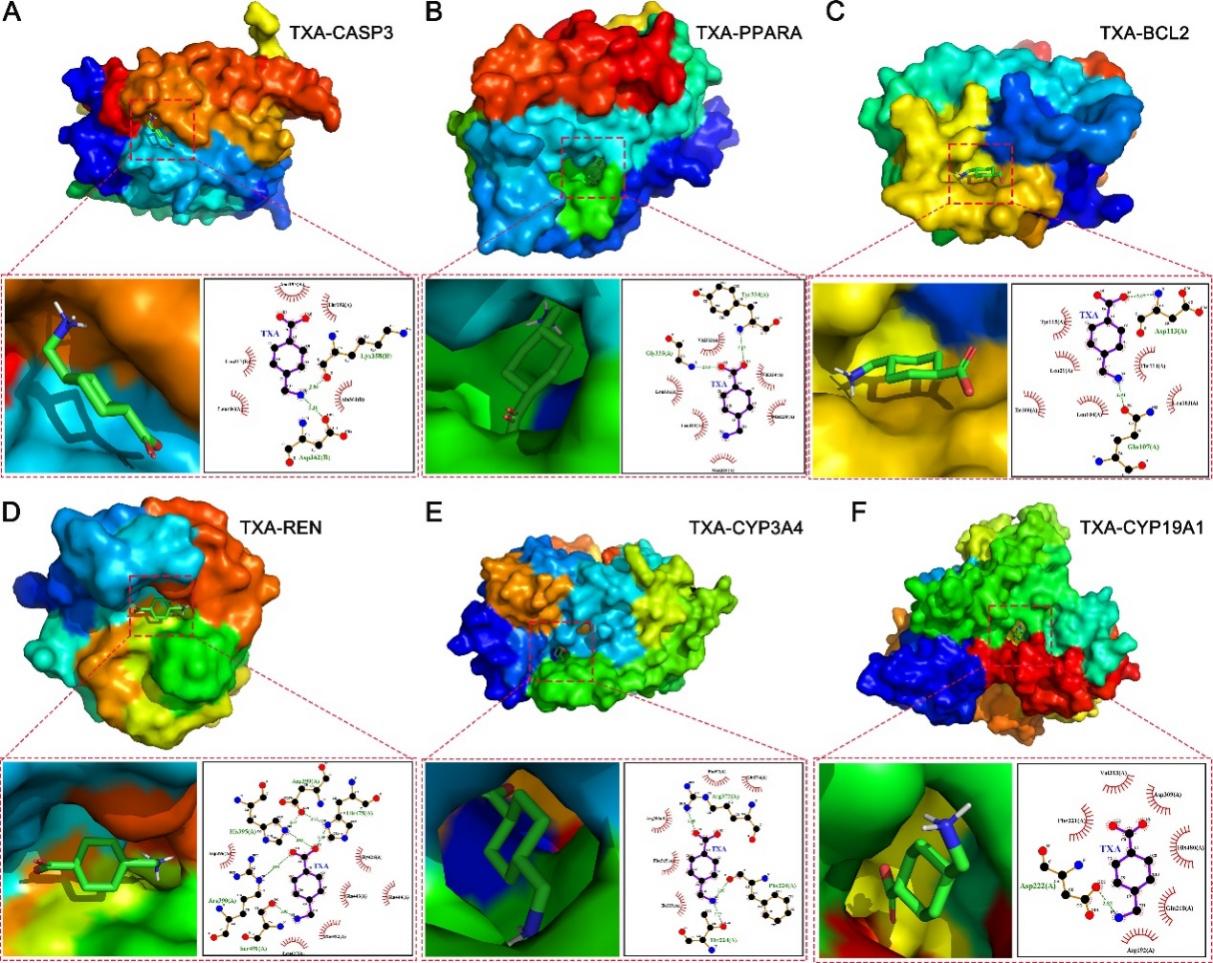
**

**Figure S4. Molecular Docking of TXA with Key Target Proteins.** (A) Schematic diagram of TXA binding to CASP3. (B) Schematic diagram of TXA binding to PPARA. (C) Schematic diagram of TXA binding to BCL2. (D) Schematic representation of TXA binding to REN. (E) Schematic representation of TXA binding to CYP3A4. (F) Schematic representation of TXA binding to CYP19A1.

**
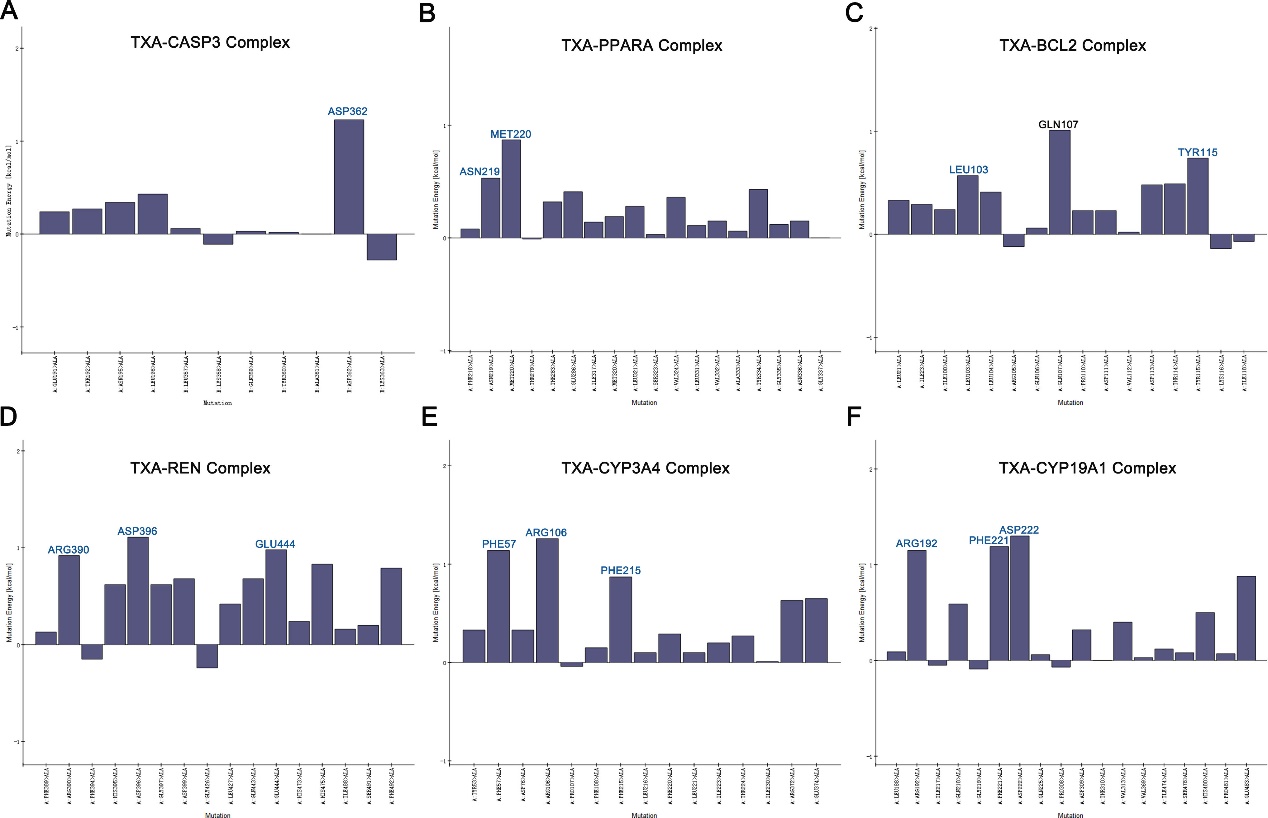
**

**Figure S5. Virtual knockout of amino acid sites in TXA-target protein complexes (within 3 Å).** (A) Virtual knockout results for the TXA-CASP3 ligand-protein complex. (B) Virtual knockout results for the TXA-PPARA ligand-protein complex. (C) Virtual knockout results for the TXA-BCL2 ligand-protein complex. (D) Virtual knockout results for the TXA-REN ligand-protein complex. (E) Virtual knockout results for the TXA-CYP3A4 ligand-protein complex. (F) Virtual knockout results for the TXA-CYP19A1 ligand-protein complex.


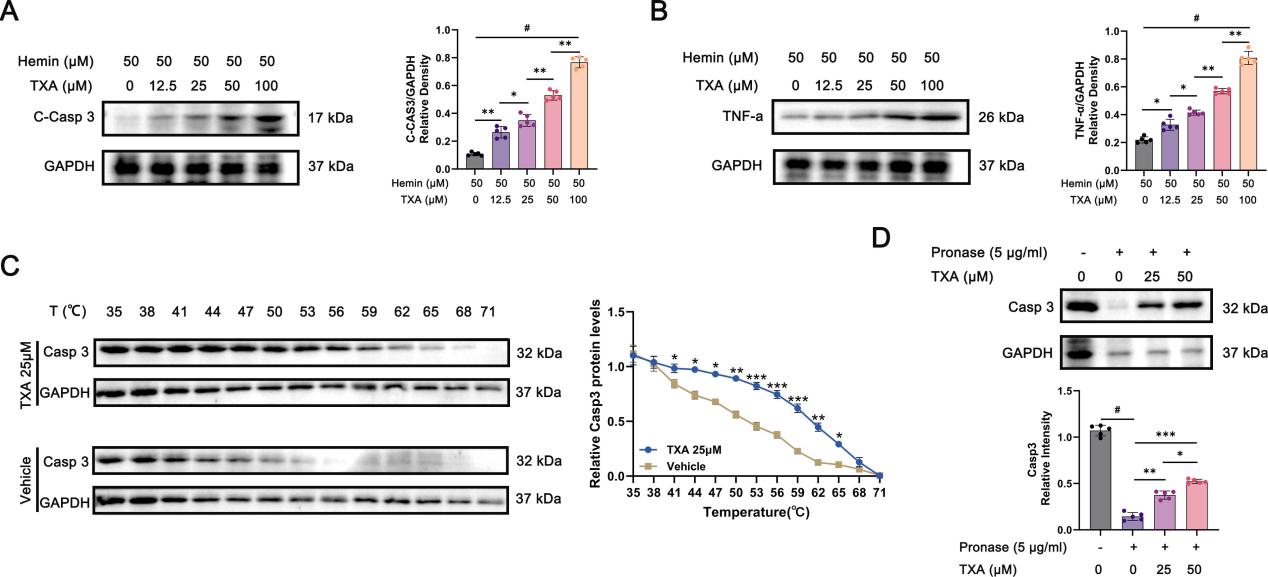


**Figure S6. Expression levels of Casp3 in HT22 and preliminary identification of target binding.** (A) Western blot analysis of C-Casp3 expression levels in HT22 cells as a function of TXA concentration. (B) Western blot analysis of TNF-α expression levels in BV2 cells as a function of TXA concentration. (C) CETSA assay used to evaluate the thermal stability of the TXA-CASP3 binding complex. (D) DARTS assay used to evaluate the hydrolytic stability of the TXA-CASP3 binding complex. All Western blot images are from independent replicate experiments and are representative of the results. All data are expressed as mean ± standard deviation (SD). Statistical significance was determined by two-way analysis of variance (ANOVA) and Tukey's multiple comparison test: * p < 0.05, ** p < 0.01, *** p < 0.001 and #p < 0.0001, n ≥ 3. ns: not significant.
